# Direct catalytic arylation of heteroarenes with *meso*-bromophenyl-substituted porphyrins

**DOI:** 10.3762/bjoc.13.152

**Published:** 2017-08-03

**Authors:** Alexei Nikolaevich Kiselev, Olga Konstantinovna Grigorova, Alexei Dmitrievich Averin, Sergei Aleksandrovich Syrbu, Oskar Iosifovich Koifman, Irina Petrovna Beletskaya

**Affiliations:** 1 G. A. Krestov Institute of Solution Chemistry RAS, Akademicheskaya ul., Ivanovo, 153045, Russia; 2 Department of Chemistry, Lomonosov Moscow State University, Leninskie Gory 1-3, Moscow, 119991, Russia; 3 Ivanovo State University of Chemistry and Technology, 7 Sheremetyevskii prosp., Ivanovo, 153000, Russia

**Keywords:** arylation, copper catalysis, heteroarenes, palladium catalysis, porphyrins

## Abstract

The arylation of mono-, di- and tetra-*meso*-bromophenyl-substituted porphyrins with the heteroarenes containing “acidic” C–H bonds, such as benzoxazole, benzothiazole and *N*-methylimidazole was studied in the presence of three alternative catalytic systems: Pd(dba)_2_/DavePhos/Cs_2_CO_3_, Pd(PPh_3_)_4_/PivOH/K_2_CO_3_ and Pd(OAc)_2_/Cu(OAc)_2_/PPh_3_/K_2_CO_3_. The first catalytic system was found to be successful in the reaction with benzoxazole, the second one was less efficient for our purpose, while the third system proved to be most versatile and afforded corresponding mono-, di-, tri- and even tetraarylated derivatives of porphyrins.

## Introduction

Porphyrins play an outstanding role in many fields of modern organic chemistry due to their unique structure and electronic properties. These molecules have already proved to be powerful tools in phototherapy, radioimmunotherapy and imaging [1], for cancer treatment [2], and actually sophisticated theranostic porphyrin-based nanodevices have been developed for guided photodynamic therapy [3]. On the other hand, various heterocycles also find multiple medical applications: bis(benzimidazoles), bis(benzoxazoles) and benzothiazoles display anticancer activities [4], 2-arylbenzothiazole is a privileged scaffold in drug discovey, and the main areas of application of such medicaments are antitumor remedies and non-invasive diagnostic imaging agents for PET, SPECT and MRI methods [5].

The combination of porphyrin and heterocyclic moieties in one molecule is perspective for the design of new compounds with potential powerful pharmaceutical properties. Up to date certain tetrapyrrole derivatives of such type were described in the literature, for example, porphyrins with imidazole and benzimidazole substitutents at *meso*-positions [6–7], *meso*-pyridinyl and *meso*-quinolinyl-substituted porphyrins [8], an imidazolyl group was attached to the *meso*-position also via acetylene bridge [9]. All these compounds were synthesized from the corresponding aldehydes already possessing an heteroaromatic moiety.

The development of the catalytic approaches opened an easy access to 2-aryl-substituted benzothiazoles, benzoxazoles and benzimidazoles. Kumada–Tamao–Corriu [10–11], Suzuki–Miyaura [12–15] and Stille couplings [16–22] were successfully applied for this purpose. Direct arylation and alkenylation are modern approaches for the formation of C–C bonds, and the application of these methodologies to porphyrins was widely studied by Osuka and co-workers. Their research was targeted at the Ir-catalyzed β-borylation of porphyrins and zinc porphyrinates with the purpose of the consequent synthesis of di- and polyporphyrin structures [23–25], further direct β-arylation of tetrapyrrolic systems with aryl bromides was developed using the Pd(OAc)_2_/DavePhos/PivOH catalytic system (DavePhos = 2-dicyclohexylphosphine-2’-dimethylaminobiphenyl, PivOH = *t-*BuCOOH) [26–27], and also bromo derivatives of condensed aromatic compounds were employed in this process [28]. The results of the Osuka group and other researchers in this field were overviewed in a short review [29]. The main features of these reactions are exclusive arylation in β-position of porphyrins and necessity to use great excesses (10–20 equiv) of aryl bromides as well as large catalyst loadings (20–30 mol % Pd(0), 40–50 mol % DavePhos). Only Ni porphyrinates were described in the arylation reactions.

In this connection we considered it important to develop an alternative approach to heteroaryl-substituted porphyrins using catalytic arylation of easily accessible *meso*-(bromophenyl)porphyrins with heterocycles possessing “acidic” C–H bonds, such as benzoxazole (p*K*_a_ 24.4), benzothiazole (p*K*_a_ 27.3), and *N*-methylbenzimidazole (p*K*_a_ 32.5) [30]. Starting mono- and di(*p*-bromophenyl)-substituted porphyrins **1** and **11** were synthesized according to a described procedure [31], their analogues **2**, **10**, **12** were obtained using essentially the same approach.

## Results and Discussion

**Scheme 1:**
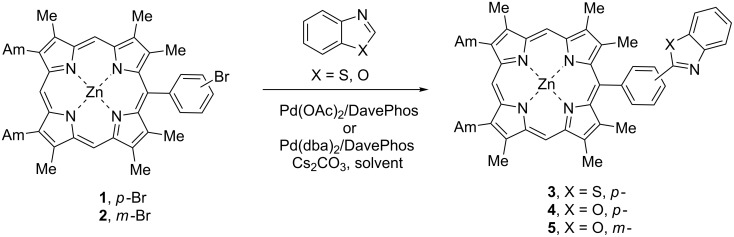
Arylation of zinc *meso*-(bromophenyl)porphyrinates **1**, **2** with benzothiazole and benzoxazole.

Zinc *meso*-(4-bromophenyl)porphyrinate (**1**) was chosen as a model substrate for the investigation of the conditions of the catalytic arylation. The reaction with benzothiazole was first catalyzed with Pd(OAc)_2_/DavePhos (2-dicyclohexylphosphino-2’-dimethylaminobiphenyl) (20:20 mol %) and carried out in DMF at 150 °C in the presence of Cs_2_CO_3_ as a base for 24 h, however, these conditions were unfavorable and provided only 8% yield of the target coupling product **3** (Scheme 1, Table 1, entry 1).

**Table 1:** Arylation of zinc *meso*-(bromophenyl)porphyrinates **1**, **2** with benzothiazole and benzoxazole (reaction time 24 h).

Entry	Porphyrin	Heterocycle	Catalytic system	Solvent, temp., °C	Product	Yield, %^a^

1	**1**	benzothiazole (1 equiv)	Pd(OAc)_2_/DavePhos (20:20 mol %)	DMF, 150	**3**	8
2	**1**	benzothiazole (2 equiv)	Pd(dba)_2_/DavePhos (20:22 mol %)	DMF, 150	**3**	32
3	**1**	benzothiazole (2 equiv)	Pd(dba)_2_/DavePhos (20:22 mol %)	dioxane, 100	**3**	34
4	**1**	benzothiazole (0.5 equiv)	Pd(dba)_2_/DavePhos (20:22 mol %)	DMF, 150	**3**	19
5	**1**	benzoxazole (1 equiv)	Pd(dba)_2_/DavePhos (20:22 mol %)	dioxane, 100	**4**	83
6	**2**	benzoxazole (2 equiv)	Pd(dba)_2_/DavePhos (20:22 mol %)	dioxane, 100	**5**	45

^a^Yields of isolated products after column chromatography.

The reaction obviously needs zerovalent palladium for the catalytic cycle and we suppose that Pd(II) was partially reduced by the phosphine ligand. The application of another catalytic system with Pd(0), Pd(dba)_2_/DavePhos (20:22 mol %, dba = dibenzylideneacetone) together with a 100% excess of benzothiazole was more successful as it afforded 32% yield of compound **3** (Table 1, entry 2); it is interesting that the use of dioxane instead of DMF and the decrease in the reaction temperature down to 100 °C, did not notably change the yield of **3** (34%, Table 1, entry 3). The use of porphyrin in excess diminished the yield (Table 1, entry 4). The reaction of benzoxazole with a more acidic C–H bond resulted in a high yield of the coupling product **4** (83%, Table 1, entry 5), though equimolar amounts of starting compounds were used. It means that the nature of the C–H bond is crucial for the result of the coupling and the nature of the catalytic system including the solvent should be adjusted to the certain pair of the reagents. Moreover, the changes in the reaction conditions may lead to the change of the mechanism of the arylation process. For example, the arylation of benzoxazole mediated by Pd(OAc)_2_/PPh_3_ presumably includes the cleavage of the 5-membered heterocycle with the formation of the transient isocyanide Pd complex followed by the ring closure [32]; on the other hand, the arylation of benzothiophene in the presence of Pd(OAc)_2_/PCy_3_*HBF_4_/PivOH proceeds in accordance with the concerted metalation–deprotonation mechanism [33–34]. We also investigated the reaction of zinc *meso*-(3-bromophenyl)porphyrinate (**2**) with a less active bromine atom in the reaction with benzoxazole (Table 1, entry 6). In this case the yield of the coupling product **5** was moderate (45%) though we employed 2 equiv of the heteroarene. The analogous reaction with benzothiazole was unsuccessful.

**Scheme 2:**
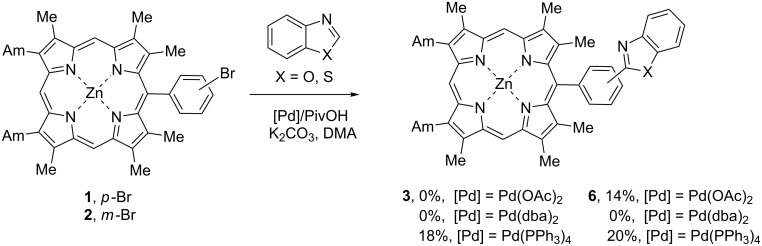
Attempts of the arylation of zinc *meso*-(bromophenyl)porphyrinates **1** and **2** with benzoxazole and benzothiazole using the Osuka protocol.

Further we decided to test the catalytic conditions proposed by Osuka for the β-arylation of porphyrins with bromoarenes [28] and investigated the reactions of the zinc porphyrinates **1** and **2** with benzoxazole (2 equiv) or benzothiazole (2 equiv) in the presence of Pd(OAc)_2_ (20 mol %) without phosphine ligand with pivalic acid as an additive (2.5 equiv). The reactions of **1** with benzoxazole and of **2** with benzothiazole were run in dimethylacetamide (DMA) at 100 °C and produced only 14% yield of the coupling product **6** with benzothiazole while with benzoxazole no reaction was observed (Scheme 2). The change of Pd(OAc)_2_ for Pd(dba)_2_ fully hindered the reaction in both cases, while the application of Pd(PPh_3_)_4_ allowed to obtain product **3** in 18% yield and slightly increased the yield of compound **6** to 20%. The accurate investigation of the mechanism of the process was not described in literature, only in the work by Hartwig [35] Pd(OAc)_2_ without any additional ligand was shown to be advantageous over catalytic systems with phosphine ligands. Osuka also used this approach for β-arylation of porphyrins. In this ligandless reaction the Pd(0) species from palladium acetate could be provided by the traces of amine present in DMA, and the catalytic cycle proceeds via Pd(II) after oxidative addition. However, Pd(dba)_2_ as a source of Pd(0) may be unfavorable in the absence of the phosphine ligand and in situ reduced palladium is sometimes more preferable. A better result obtained with Pd(PPh_3_)_4_ supports this consideration.

**Scheme 3:**
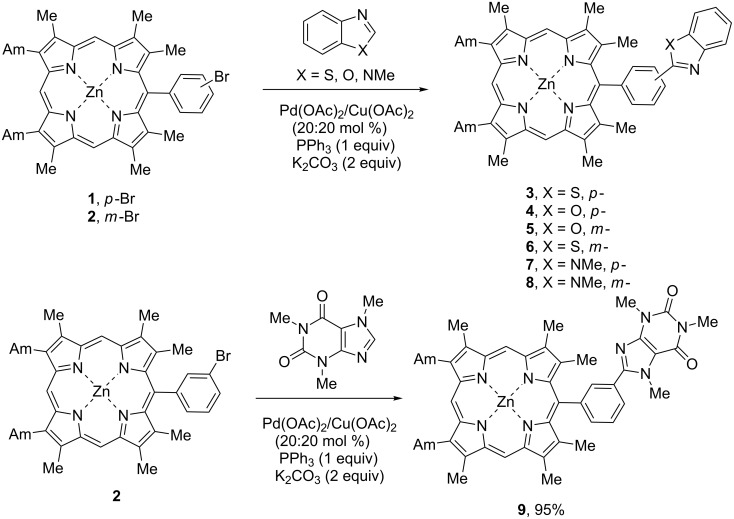
Arylation of zinc *meso*-(bromophenyl)porphyrinates **1**, **2** with benzothiazole, benzoxazole, *N*-methylbenzimidazole and caffeine.

In our further investigations we abandoned this catalytic protocol which was shown to be improper for our reagents and tried another one, proposed by Z.-Z. Huang [36] which employs both Pd(II) and Cu(II) catalyst precursors. The reactions of zinc porphyrinates **1** and **2** with heteroarenes were catalyzed with Pd(OAc)_2_/Cu(OAc)_2_ (20:20 mol %) catalytic system in the presence of 1 equiv PPh_3_ ligand and K_2_CO_3_ as a base (Scheme 3). All reactions were run in boiling toluene for 24 h. At first the ratio of porphyrin **1** to heteroarene was taken as 1:0.9 (Table 2, entries 1, 3, and 6) and with benzothiazole and benzoxazole it provided moderate yields of the target compounds **3** and **4** (35 and 47%, respectively). In the reaction with *N*-methylbenzimidazole no coupling product could be isolated in a pure state due to the formation of the complex reaction mixture and insufficient conversion of the starting compounds. The application of 2 equiv of heteroarenes like in protocols described above led to a notable improvement of the yields which attained 60% for the compounds **3** and **4** (Table 2, entries 2 and 4), and we also managed to isolate an individual coupling product with *N*-methylbenzimidazole (Table 2, entry 7). The increase in the reaction time (40 h instead of 24 h) was not significant for the arylation with benzoxazole (Table 2, entry 5) but it helped to obtain compound **7** in a high yield (Table 2, entry 8). With benzothiazole the increase in the reaction time led not only to a higher conversion of the starting compounds but also to a substantial destruction of the reaction product. This effect explains inefficiency of the longer reaction time for the synthesis of product **3**.

**Table 2:** Arylation of zinc *meso*-(bromophenyl)porphyrinates **1**, **2** with benzothiazole, benzoxazole, *N*-methylbenzimidazole and caffeine (conditions: Pd(OAc)_2_/Cu(OAc)_2_ (20:20 mol %), PPh_3_, 1 equiv, K_2_CO_3_, toluene, 110 °C).

Entry	Porphyrin	Heterocycle	Porphyrin to heterocycle ratio	Time, h	Product	Yield, %

1	**1**	benzothiazole	1:0.9	24	**3**	35
2	**1**	benzothiazole	1:2	24	**3**	60
3	**1**	benzoxazole	1:0.9	24	**4**	47
4	**1**	benzoxazole	1:2	24	**4**	60
5	**1**	benzoxazole	1:2	40	**4**	67
6	**1**	*N*-methylbenzimidazole	1:0.9	24	**7**	0
7	**1**	*N*-methylbenzimidazole	1:2	24	**7**	29
8	**1**	*N*-methylbenzimidazole	1:2	40	**7**	79
9	**2**	benzothiazole	1:2	40	**6**	55
10	**2**	benzoxazole	1:2	40	**5**	50
11	**2**	*N*-methylbenzimidazole	1:2	40	**8**	90
12	**2**	caffeine	1:2	40	**9**	95

The reactions with a less reactive *meso*-(3-bromophenyl)porphyrin **2** were run for 40 h using 2 equiv of heteroarenes and provided 50–55% yields of the corresponding coupling products with benzoxazole and benzothiazole (Table 2, entries 9 and 10), and with *N*-methylbenzimidazole the yield of **8** reached 90% (Table 2, entry 11). However, the best result was obtained with caffeine (95%, Table 2, entry 12) which turned to be the most active reagent in the series.

**Scheme 4:**
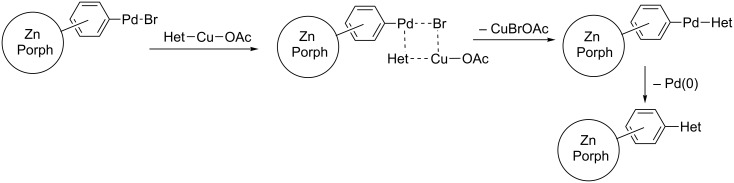
Plausible action of palladium and copper catalysts with transmetalation step.

The mechanism of this catalytic process is still not clear. Indeed, Cu(II) which is present in an equimolar amount compared to Pd(II) should oxidize zero valent palladium emerging in the course of the reduction with excess PPh_3_ (like in the Wacker process). A plausible explanation is that Cu(II) metalates heteroarene in the presence of K_2_CO_3_ forming Het-Cu-OAc species and this process proceeds faster than the oxidation of Pd(0). Then one may suppose the transmetalation reaction at heteroarene according to Scheme 4 which provides the formation of Ar-Pd-Het intermediate giving the target coupling product. Probably this path is better than direct palladation of heteroarenes. As a result, the needed excess of heteroarene is dramatically decreased (from 10–20 equiv used by Osuka to 2 equiv in the present protocol).

**Scheme 5:**
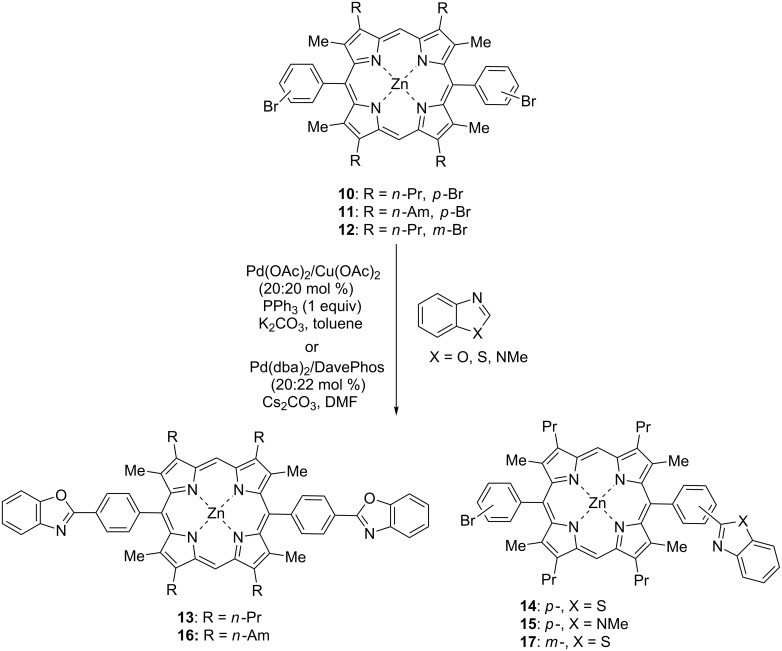
Dirylation of zinc di-*meso*-(bromophenyl)porphyrinates **10–12** with benzothiazole, benzoxazole and *N*-methyl benzimidazole.

At the next step of our investigation we employed zinc di-*meso*-(bromophenyl)porphyrinates **10–12** in the reactions with the same heteroarenes to compare two catalytic systems (Scheme 5, Table 3). The reaction were run under optimized conditions for 24 h in the case of benzothiazole and 40 h in the case of benzoxazole and *N*-methylbenzimidazole using 4 equiv of heteroarenes, the amounts of the catalysts were taken as described above. The reactions with these porphyrins turned to be more capricious and the results depended seriously on the nature of both reagents. The arylation of porphyrin **10** with benzoxazole in the presence of Pd(II)/Cu(II) catalytic system in toluene provided a diarylated product **13** in a good yield (65%, Table 3, entry 1). However, a less reactive benzothiazole gave only monoarylated product **14** in 64% yield (Table 3, entry 2). Surprisingly, in the case of *N*-methylbenzimidazole we achieved 83% yield of the monoarylated product **15**, but no target diarylated product was obtained (Table 3, entry 3). Alternative catalytic system Pd(dba)_2_/DavePhos proved to be efficient for the synthesis of the diarylated product **16** in the reaction of benzoxazole with porphyrin **11** (Table 3, entry 4) which is quite similar to porphyrin **10** (it contains *n*-amyl substitutents instead of *n*-propyl groups), however, this catalytic system was unable to provide the arylation with benzothiazole or *N*-benzimidazole under the same conditions.

**Table 3:** Diarylation of zinc di-*meso*-(bromophenyl)porphyrinates **10–12** with benzothiazole, benzoxazole and *N*-methylbenzimidazole.

Entry	Porphyrin	Heterocycle	Catalytic system	Type of substitution	Product	Yield, %

1	**10**	benzoxazole	Pd(OAc)_2_/Cu(OAc)_2_/PPh_3_	disubstitution	**13**	65
2	**10**	benzothiazole	Pd(OAc)_2_/Cu(OAc)_2_/PPh_3_	monosubstitution	**14**	64
3	**10**	*N*-methylbenzimidazole	Pd(OAc)_2_/Cu(OAc)_2_/PPh_3_	monosubstitution	**15**	83
4	**11**	benzoxazole	Pd(dba)_2_/DavePhos	disubstitution	**16**	52
5	**12**	benzothiazole	Pd(OAc)_2_/Cu(OAc)_2_/PPh_3_	monosubstitution	**17**	19

Isomeric zinc di-*meso*-(3-bromophenyl)porphyrinate **12** was able to form only monoarylated derivative **17** from benzothiazole in a low yield (Table 3, entry 5) in the presence of the catalytic system Pd(OAc)_2_/Cu(OAc)_2_/PPh_3_ but could not provide the arylation products with either benzoxazole or *N*-methylbenzimidazole. These facts imply that one cannot judge the reactivity of any hetoeroarene itself but need to refer to a pair of reagents also indicating a certain catalytic system in each case.

**Scheme 6:**
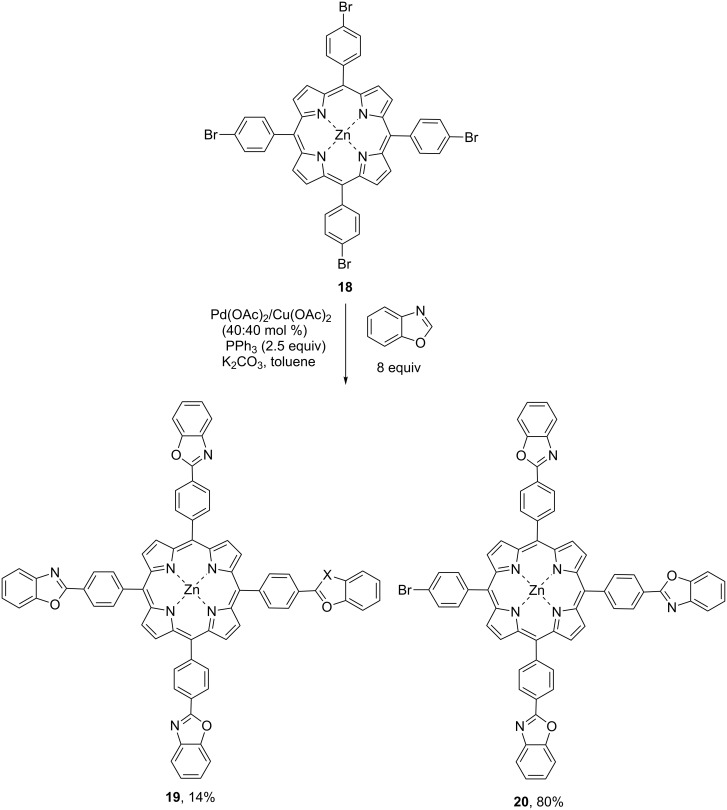
Polyarylation of zinc tetrakis-*meso*-(bromophenyl)porphyrinate **18** with benzoxazole.

In order to explore the scope and limitations of the studied approach we carried out the reaction with zinc tetrakis(4-bromophenyl)porphyrinate **18** using 8 equiv of the corresponding heteroarenes, 40 mol % catalyst, 2.5 equiv PPh_3_ and 5 equiv K_2_CO_3_ (Scheme 6). It was found out that with benzoxazole the target tetraarylated derivative **19** was isolated in 14% yield, and triarylated compound **20** was the major product obtained in 80% yield. With benzothiazole and *N*-benzimidazole the same reactions provided only inseparable mixtures of corresponding tri- and tatraarylated derivatives.

The investigation of the UV–vis spectra of the arylated monophenylporphyrins **3–9** disclosed that they possess almost identical absorption maxima but somewhat differ in extinction values primarily due to the different sharpness of the bands (Table 4). The Soret band at 406 nm is naturally the most intensive while Q bands at 535 and 570 nm are almost of the same intensities. Thus the maxima of the absorption bands in the products **3–9** are essentially the same as in the starting Zn porphyrinates **1** (λ_max_ 572 nm (lgε 3.95), 536 nm (lgε 3.98), 406 nm (lgε 5.56)) [31] and **2** (571 nm (lgε 4.42), 534 nm (lgε 4.43), 405 nm (lgε 5.64)), but extinction values are regularly smaller. The spectra of fluorescence (with extinction at Soret band) in all cases display two emission maxima at 586 and 636–638 nm, the first one being more intensive. It corresponds with the fluorescence spectra of zinc bromophenylporphyrinate.

**Table 4:** UV–vis and fluorescence spectra of the arylated porphyrins **3–9**, **13–15** (in CH_2_Cl_2_).

Compound	λ_max_, nm	lgε	λ_em_, nm

**3**	406 535 570	4.59 2.94 2.80	586 636
**4**	406 535 571	4.89 3.49 3.47	586 636
**5**	406 535 571	4.58 3.38 3.40	586 636
**6**	406 535 571	4.84 3.47 3.44	586 636
**7**	406 535 571	4.84 3.28 3.23	586 638
**8**	406 535 571	4.50 3.30 3.26	586 636
**9**	406 533 571	4.42 1.94 2.08	586 636
**13**	413 539 574	4.93 3.52 3.10	592 642
**14**	410 538 573	4.50 2.91 2.21	592 640
**15**	410 536 572	4.70 2.93 2.13	592 642

The UV–vis spectra of diphenylporphyrins **13–15** (Table 4) possess absorption bands in the same region as their monophenyl analogues **3**–**9**, but all maxima are slightly red-shifted. It fully corresponds with the absorption bands of the bis(4-bromophenyl)porphyrin **11** (λ_max_ 575 (lgε 3.16), 540 (lgε 3.38), 412 (lgε 4.68)). There is no significant difference in the spectra of diarylated derivative **13** and monoarylated derivatives **14** and **15**, one may note a slight bathochromic shift of the Soret band and higher extinction values for compound **13**. The spectra of fluorescence are quite identical and are characterized by two emission bands at 592 and 640–642 nm. All these spectroscopic data demonstrate that the substitution of the bromine atom for heterocyclic moieties does not notably change absorption and emission spectra due to insignificant influence of the electronic properties of the substituents at the *meso*-phenyl rings on the electronic structure of the porphyrin core.

## Conclusion

To sum up, the investigation of the arylation of zinc *meso*-(bromophenyl)porphyrinates under different catalytic conditions revealed that the catalytic system Pd(OAc)_2_/Cu(OAc)_2_/PPh_3_ was the most efficient and applicable to various pairs of the reagents. More successful arylation of 4-bromophenyl derivatives compared to 3-bromophenyl-substituted porphyrinates is due to a more reactive halogen in these compounds. Generally, benzoxazole and in some cases *N*-methylbenzimidazole demonstrated the advantage over benzothiazole in the catalytic coupling probably due to lower stability of the latter under the reaction conditions studied. As a result, the possibility of successful diarylation of zinc di-*meso*-(4-bromophenyl)porphyrinate and even tetraarylation of zinc tetrakis-*meso*-(bromophenyl)porphyrinate with benzoxazole was shown.

## Supporting Information

File 1 Experimental procedures, characterization and spectral data for synthesized compounds **2–9**, **11, 12, 13–17, 19, 20**.
