# Supplementary material for: Direct catalytic arylation of heteroarenes with meso-bromophenyl-substituted porphyrins
Source: Beilstein J Org Chem. 2017 Aug 3;13:1524–32. doi: 10.3762/bjoc.13.152 (PMC5550796; doi:10.3762/bjoc.13.152)
Supplement: File 1 — Experimental procedures, characterization and spectral data for synthesized compounds 2–9, 11, 12, 13–17, 19, 20. [file Beilstein_J_Org_Chem-13-1524-s001.pdf]

Supporting Information

for

Direct catalytic arylation of heteroarenes with *meso*-bromophenyl-substituted porphyrins

Alexei N. Kiselev[1], Olga K. Grigorova[2], Alexei D. Averin[2], Sergei A. Syrbu[1], Oskar I. Koifman[3] and Irina P. Beletskaya[2]*

Address: [1]G. A. Krestov Institute of Solution Chemistry RAS, 1 Akademicheskaya ul., Ivanovo, 153045, Russia, [2]Department of Chemistry, Lomonosov Moscow State University, Leninskie Gory 1-3, Moscow, 119991, Russia and [3]Ivanovo State University of Chemistry and Technology, 7 Sheremetyevskii prosp., Ivanovo, 153000, Russia

Email: Irina P. Beletskaya* - beletska@org.chem.msu.ru

*Corresponding author

Experimental procedures, characterization and spectral data for synthesized compounds 2–9, 11, 12, 13–17, 19, 20.

Experimental part

General

NMR spectra were recorded with Bruker Avance-400 spectrometer (^1H at 400 MHz, ^{13}C at 100.6 MHz) in $CDCl_3$ at 298 K. Chemical shifts are given in δ scale in ppm, signals in proton spectra are referenced to residual peaks of $CHCl_3$ (δ_H 7.25), signals in ^{13}C spectra are referenced to the centers of multiplets of $CDCl_3$ (δ_c 77.0). MALDI-TOF mass spectra of positive ions were registered with Bruker Daltonics Autoflex II and Shimadzu Axima Confidence spectrometers. UV-vis spectra were recorded with Agilent Cary 60 spectrometer in CH_2Cl_2, fluorescence spectra were obtained using Horiba Jobin Yvon Fluoromax-2 spectrometer in CH_2Cl_2. Preparative column chromatography was carried out using silica gel Merck 40-60 mesh.

Commercially available benzoxazole, benzothiazole, *N*-methylbenzimidazole, palladium acetate, copper (II) acetate dihydrate, potassium carbonate, cesium carbonate, triphenylphosphine, 2-dicyclohexylphosphino-2’-dimethylaminobiphenyl (DavePhos), pivalic acid,

tetrakis(triphenylphosphine) palladium were used without special purification. $Pd(dba)_2$ was obtained according to a described method [1]. Tetrakis(4-bromophenyl)porphyrin for making its Zn complex **18** was synthesized as described in [2]. Dimethylformamide and dimethylacetamide were distilled over CaH_2, dioxane and toluene were distilled over sodium, dichloromethane, hexanes and methanol were used freshly distilled.

Zinc 5-(3-bromophenyl)-2,3,7,8,12,18-hexamethyl-13,17-di(*n*-pentyl)porphyrinate (**2**) was obtained by the reaction with excess zinc acetate of the corresponding free base which in turn was synthesized in accordance with the method described for its isomer **1** [3]. The free base was obtained in 15% yield, and the Zn complex was synthesized in 99% yield. Dark-red crystalline powder. UV/vis (CH_2Cl_2) λ_{max}, nm (lgε) 571 (4.42), 534 (4.43), 405 (5.64) nm. ^{1}H NMR ($CDCl_3$) δ 0.95 (t, 6H, ^3J = 7.3 Hz), 1.48 (sextet, 4H, ^3J = 7.2 Hz), 1.62 (quintet, 4H, ^3J = 7.4 Hz), 2.01 (quintet, 4H, ^3J = 6.8 Hz), 2.45 (br.s, 6H), 3.33 (br.s, 6H), 3.44 (br.s, 6H), 3.50 (br.s, 4H), 7.61 (t, 1H, ^3J = 7.7 Hz), 7.93 (br.d, 1H, $^3J_{obs}$ = 6.9 Hz), 7.97 (d, 1H, ^3J = 8.0 Hz), 8.28 (s, 1H), 9.03 (br.s, 1H), 9.69 (br.s, 2H); MS (MALDI-TOF) calcd. for $C_{42}H_{47}BrN_4Zn$: 750.23, found 750.37. $[M]^+$.

Zinc 5,15-bis(4-bromophenyl)-3,7,13,17-tetramethyl-2,8,12,18-tetra(*n*-propyl)porphyrinate (**10**) was obtained by the reaction with excess zinc acetate of the corresponding free base which in turn was synthesized in accordance with the method described for its analogue **11** [3]. The free base was obtained in 18% yield, and the Zn complex was synthesized in 98% yield. Dark-red crystalline powder. UV/vis (CH_2Cl_2) λ_{max}, nm (lgε) 574 (4.42), 538 (4.54), 410 (5.67). ^{1}H NMR ($CDCl_3$) δ 1.27 (t, 12H, ^3J = 7.3 Hz), 2.20 (sextet, 8H, ^3J = 7.3 Hz), 2.49 (s, 12H), 3.95 (t, 8H, ^3J = 7.3 Hz), 7.88 (d, 4H, ^3J = 7.7 Hz), 7.97 (d, 4H, ^3J = 7.7 Hz), 10.18 (s, 2H); MS (MALDI-TOF) calcd. for $C_{48}H_{50}Br_2N_4Zn$: 904.17, found 904.92. $[M]^+$.

Zinc 5,15-bis(3-bromophenyl)-3,7,13,17-tetramethyl-2,8,12,18-tetra(*n*-propyl)porphyrinate (**12**) was obtained by the reaction with excess zinc acetate of the corresponding free base which in turn was synthesized in accordance with the method described for its analogue **11** [3]. The free base was obtained in 18% yield, and Zn complex was synthesized in 98% yield. Dark-red crystalline powder. UV/vis (CH_2Cl_2) λ_{max}, nm (lge) 576 (4.34), 541 (4.51), 411 (5.65). ^{1}H NMR ($CDCl_3$) δ 1.27 (t, 12H, ^3J = 7.1 Hz), 2.22 (sextet, 8H, ^3J = 7.1 Hz), 2.50 (s, 12H), 3.95 (br.s, 8H), 7.61 (t, 2H, ^3J = 7.7 Hz), 7.95 (d, 2H, ^3J = 8.0 Hz), 8.02 (br.d, 2H, $^3J_{obs}$ = 5.8 Hz), 8.27 (s, 2H), 10.19 (s, 2H); MS (MALDI-TOF) calcd. for $C_{48}H_{50}Br_2N_4Zn$: 904.17, found 904.89. $[M]^+$.

General method for Pd(0)-catalyzed arylation of heteroarenes.

A Schlenk tube equipped with a magnetic stirrer and reflux condenser was flushed with dry argon, charged with $Pd(dba)_2$ or $Pd(OAc)_2$ (20 mol %), phosphine ligand DavePhos (20 mol % in the case of $Pd(OAc)_2$, 22 mol % in the case of $Pd(dba)_2$), appropriate porphyrin (0.05 mmol), then absolute dioxane (1.5 ml) or DMF (1.5 ml) was added followed by a corresponding heteroarene (0.05–0.1 mmol in the case of monoarylation or 0.125 mmol in the case of diarylation). The reaction mixture was stirred for 1 min, then cesium carbonate (0.1 mmol, 33 mg) was added and the reaction was stirred under reflux at 100 °C in the case of dioxane or at 150 °C in the case of DMF. After ca. 40 h upon completion of the reaction the mixture was cooled down to ambient temperature, dichloromethane (5 ml) was added, the organic solution was filtered, the residue was washed additionally with dichloromethane (2 × 5 ml), combined organic fractions were evaporated in vacuo. To obtain individual compounds, the residue was chromatographed on silica gel using a sequence of eluents: hexanes/CH_2Cl_2 (1:1), CH_2Cl_2, CH_2Cl_2/MeOH (100:1).

General method for Pd(II)/Cu(II)-catalyzed arylation of heteroarenes

A Schlenk tube equipped with a magnetic stirrer and reflux condenser, was charged with $Pd(OAc)_2$ (20-40 mol%), $Cu(OAc)_2 \cdot 2H_2O$ (20–40 mol %), triphenylphosphine (1 equiv), corresponding porphyrin (generally 0.05 mmol), toluene (3 ml), appropriate heteroarene (generally 0.1 mmol for the monoarylation processes, 0.2 mmol for duarylation processes and 0.4 mmol for tetraaryaltion process). The reaction mixture was stirred for 1 min, then potassium carbonate (0.1–0.25 mmol) was added and the reaction mixture was stirred under reflux at 110 °C. After ca. 24 h upon completion of the reaction the mixture was cooled down to ambient temperature, dichloromethane (5 ml) was added, the organic solution was filtered, the residue was washed additionally with dichloromethane (2 × 5 ml), combined organic fractions were evaporated in vacuo. To obtain individual compounds, the residue was chromatographed on silica gel using a sequence of eluents: hexanes/CH_2Cl_2 (1:1), CH_2Cl_2, CH_2Cl_2/MeOH (100:1), CH_2Cl_2/MeOH (50:1).

Zn (II) complex of 12-[4-(1,3-benzothiazol-2-yl)phenyl]-5,9,10,14,15,19-hexamethyl-4,20-dipentyl-21,22,23,24-tetraazapentacyclo[16.2.1.1^{3,6}.1^{8,11}.1^{13,16}]tetracosa-1(21),2,4,6,8(23),9,11,13,15,17,19-undecaene (**3**) was obtained from porphyrin **1** (0.05 mmol, 38 mg), benzothiazole (0.1 mmol, 11 mg (12 μl)) in the presence of $Pd(OAc)_2$ (2.5 mg), $Cu(OAc)_2 \cdot 2H_2O$ (2 mg), PPh_3 (13 mg) and K_2CO_3 (14 mg) in 3 ml toluene. Eluent CH_2Cl_2. Yield 24 mg (60%), dark-red crystalline powder. ^{1}H NMR ($CDCl_3$) δ 0.97 (t, 6H, ^3J = 7.2 Hz),

1.51 (sextet, 4H, ^3J = 7.4 Hz), 1.68 (quintet, 4H, ^3J = 7.4 Hz), 2.14 (quintet, 4H, ^3J = 7.4 Hz), 2.46 (s, 6H), 3.44 (s, 12H), 3.72 (t, 4H, ^3J = 7.6 Hz), 7.51-7.55 (m, 1H), 7.57-7.61 (m, 1H), 7.95 (d, 1H, ^3J = 8.3 Hz), 8.12 (d, 2H, ^3J = 8.1 Hz), 8.22 (d, 1H, ^3J = 8.2 Hz), 8.48 (d, 2H, ^3J = 8.1 Hz), 9.43 (s, 1H), 9.85 (s, 2H); MS (MALDI-TOF) calcd. for $C_{49}H_{51}N_5SZn$: 805.316, found 805.317. $[M]^+$.

Zn complex of 12-[4-(1,3-benzoxazol-2-yl)phenyl]-5,9,10,14,15,19-hexamethyl-4,20-dipentyl-21,22,23,24-tetraazapentacyclo[16.2.1.1^{3,6}.1^{8,11}.1^{13,16}]tetracosa-1(21),2,4,6,8(23),9,11,13,15,17,19-undecaene (4) was obtained from porphyrin **1** (0.05 mmol, 38 mg), benzoxazole (0.05 mmol, 6 mg) in the presence of $Pd(dba)_2$ (6 mg), DavePhos (4 mg), and Cs_2CO_3 (33 mg) in 1.5 ml dioxane. Eluent CH_2Cl_2. Yield 31 mg (83%), dark-red crystalline powder. ^{1}H NMR ($CDCl_3$) δ 0.97 (t, 6H, ^3J = 7.2 Hz), 1.53 (sextet, 4H, ^3J = 7.2 Hz), 1.68 (quintet, 4H, ^3J = 7.4 Hz), 2.15 (quintet, 4H, ^3J = 7.1 Hz), 2.45 (br.s, 6H), 3.47 (br.s, 12H), 3.74 (br.s, 4H), 7.32-7.37 (m, 2H), 7.50-7.53 (m, 1H), 7.75-7.78 (m, 1H), 8.21 (d, 2H, ^3J = 7.7 Hz), 8.66 (d, 2H, ^3J = 7.7 Hz), 9.46 (br.s, 1H), 9.87 (br.s, 2H); MS (MALDI-TOF) calcd. for $C_{49}H_{51}N_5OZn$: 789.339, found 789.348. $[M]^+$.

Zn complex of 12-[3-(1,3-benzoxazol-2-yl)phenyl]-5,9,10,14,15,19-hexamethyl-4,20-dipentyl-21,22,23,24-tetraazapentacyclo[16.2.1.1^{3,6}.1^{8,11}.1^{13,16}]tetracosa-1(21),2,4,6,8(23),9,11,13,15,17,19-undecaene (5) was obtained from porphyrin **2** (0.05 mmol, 38 mg), benzoxazole (0.1 mmol, 12 mg) in the presence of $Pd(OAc)_2$ (2.5 mg), $Cu(OAc)_2 \cdot 2H_2O$ (2 mg), PPh_3 (13 mg) and K_2CO_3 (14 mg) in 3 ml toluene. Eluent CH_2Cl_2. Yield 20 mg (50%), dark-red crystalline powder. ^{1}H NMR ($CDCl_3$) δ 0.94 (t, 6H, ^3J = 7.3 Hz), 1.49 (sextet, 4H, ^3J = 7.3 Hz), 1.64 (quintet, 4H, ^3J = 7.3 Hz), 2.07 (quintet, 4H, ^3J = 7.0 Hz), 2.49 (br.s, 6H), 3.44 (br.s, 12H), 3.61 (br.s, 4H), 7.30-7.36 (m, 2H), 7.52 (d, 1H, ^3J = 7.3 Hz), 7.79 (d, 1H, ^3J = 8.1 Hz), 7.94 (t, 1H, ^3J = 7.6 Hz), 8.22 (d, 1H, ^3J = 7.1 Hz), 8.75 (d, 1H, ^3J = 7.8 Hz), 9.04 (s, 1H), 9.19 (br.s, 1H), 9.77 (br.s 2H); ^{13}C NMR ($CDCl_3$) δ 11.4 (br, 2C), 12.2 (br, 2C), 14.1 (2C), 15.9 (br, 2C), 22.7 (2C), 26.2 (br, 2C), 32.3 (2C), 33.0 (br, 2C), 96.8 (br, 3C), 110.6, 120.1, 124.6, 125.2, 126.3, 127.3, 128.1, 132.2 (br), 136.0 (br), 137.0-138.5 (br.m), 140.7 (br), 142.2, 145.0 (br), 145.5-148.0 (br.m), 150.9, 163.3; aromatic carbon atoms were not integrated due to line broadening and overlapping of some signals; MS (MALDI-TOF) calcd. for $C_{49}H_{51}N_5OZn$: 789.339, found 789.323. $[M]^+$.

Zn complex of 12-[3-(1,3-benzothiazol-2-yl)phenyl]-5,9,10,14,15,19-hexamethyl-4,20-dipentyl-21,22,23,24-tetraazapentacyclo[16.2.1.1^{3,6}.1^{8,11}.1^{13,16}]tetracosa-

1(21),2,4,6,8(23),9,11,13,15,17,19-undecaene (6) was obtained from porphyrin **2** (0.05 mmol, 38 mg), benzothiazole (0.1 mmol, 11 mg (12 μl)) in the presence of $Pd(OAc)_2$ (2.5 mg), $Cu(OAc)_2 \cdot 2H_2O$ (2 mg), PPh_3 (13 mg) and K_2CO_3 (14 mg) in 3 ml toluene. Eluent CH_2Cl_2. Yield 22 mg (55%), dark-red crystalline powder. ^1H NMR ($CDCl_3$) δ 0.94 (t, 6H, ^3J = 7.1 Hz), 1.49 (sextet, 4H, ^3J = 7.4 Hz), 1.63 (quintet, 4H, ^3J = 7.2 Hz), 2.04 (quintet, 4H, ^3J = 7.2 Hz), 2.51 (s, 6H), 3.35 (s, 6H), 3.46 (s, 6H), 3.56 (br.s, 4H), 7.42-7.53 (m, 2H), 7.86 (d, 1H, ^3J = 8.1 Hz), 7.91 (t, 1H, ^3J = 7.3 Hz), 8.06 (d, 1H, ^3J = 8.6 Hz), 8.16 (br.d, 1H, $^3J_{obs}$ = 6.7 Hz), 8.62 (d, 1H, ^3J = 7.5 Hz), 8.87 (s, 1H), 9.08 (s, 1H), 9.72 (s 2H); ^{13}C NMR ($CDCl_3$) δ 11.4 (2C), 12.1 (2C), 14.1 (2C), 16.0 (2C), 22.7 (2C), 26.2 (2C), 32.3 (2C), 32.8 (2C), 96.0 (1C), 97.0 (2C), 121.6, 121.9, 123.3, 124.0, 125.2, 126.4, 126.5, 126.7, 127.0, 128.1, 132.2, 132.8, 135.1, 135.6, 137.6, 138.3, 140.9, 145.1, 146.7, 147.5, 154.3, 168.2; aromatic carbon atoms were not integrated due to ambiguity in their assignment and eventual overlapping of some signals; MS (MALDI-TOF) calcd. for $C_{49}H_{51}N_5SZn$: 805.32, found 805.22. $[M]^+$.

Zn complex of 5,9,10,14,15,19-hexamethyl-12-[4-(1-methyl-1*H*-benzimidazol-2-yl)phenyl]-4,20-dipentyl-21,22,23,24-tetraazapentacyclo[16.2.1.1^{3,6}.1^{8,11}.1^{13,16}]tetracosa-1(21),2,4,6,8(23),9,11,13,15,17,19-undecaene (7) was obtained from porphyrin **1** (0.05 mmol, 38 mg), 1-methylbenzimidazole (0.1 mmol, 13 mg) in the presence of $Pd(OAc)_2$ (2.5 mg), $Cu(OAc)_2 \cdot 2H_2O$ (2 mg), PPh_3 (13 mg) and K_2CO_3 (14 mg) in 3 ml toluene. Eluent CH_2Cl_2. Yield 31 mg (79%), dark-red crystalline powder. ^1H NMR ($CDCl_3$) δ 0.97 (t, 6H, ^3J = 7.2 Hz), 1.53 (sextet, 4H, ^3J = 7.2 Hz), 1.70 (quintet, 4H, ^3J = 7.3 Hz), 2.19 (quintet, 4H, ^3J = 6.8 Hz), 2.49 (s, 3H), 3.48 (s, 6H), 3.49 (s, 6H), 3.82 (t, 4H, ^3J = 7.4 Hz), 4.15 (s, 3H), 7.41-7.46 (m, 2H), 7.52-7.55 (m, 1H), 7.98-8.01 (m, 1H), 8.15 (d, 2H, ^3J = 7.6 Hz), 8.21 (d, 2H, ^3J = 7.6 Hz), 9.62 (s, 1H), 9.94 (s, 2H); ^{13}C NMR ($CDCl_3$) δ 11.6 (2C), 12.1 (2C), 14.2 (2C), 15.7 (2C), 22.8 (2C), 26.4 (2C), 32.2 (1C), 32.3 (2C), 32.9 (2C), 96.6 (1C), 97.3 (2C), 109.8, 119.9, 122.8, 123.1, 128.5, 129.8, 133.6, 136.1, 137.7, 138.4, 141.3, 145.9, 146.6, 147.0, 148.0, 148.1, 153.8; aromatic carbon atoms were not integrated due to ambiguity in their assignment and eventual overlapping of some signals; MS (MALDI-TOF) calcd. for $C_{50}H_{54}N_6Zn$: 802.370, found 802.377. $[M]^+$.

Zn complex of 5,9,10,14,15,19-hexamethyl-12-[3-(1-methyl-1*H*-benzimidazol-2-yl)phenyl]-4,20-dipentyl-21,22,23,24-tetraazapentacyclo[16.2.1.1^{3,6}.1^{8,11}.1^{13,16}]tetracosa-1(21),2,4,6,8(23),9,11,13,15,17,19-undecaene (8) was obtained from porphyrin **2** (0.05 mmol, 38 mg), *N*-methylbenzimidazole (0.1 mmol, 13 mg) in the presence of $Pd(OAc)_2$ (2.5 mg), $Cu(OAc)_2 \cdot 2H_2O$ (2 mg), PPh_3 (13 mg) and K_2CO_3 (14 mg) in 3 ml toluene. Eluent CH_2Cl_2.

Yield 36 mg (90%), dark-red crystalline powder. ^{1}H NMR ($CDCl_3$) δ 0.97 (t, 6H, ^3J = 7.3 Hz), 1.48 (sextet, 4H, ^3J = 7.2 Hz), 1.63 (quintet, 4H, ^3J = 7.3 Hz), 2.06 (br.s, 4H), 2.48 (s, 6H), 3.36 (s, 6H), 3.43 (s, 6H), 3.58 (br.s, 4H), 3.93 (s, 3H), 7.26-7.35 (m, 3H), 7.87 (d, 1H, ^3J = 8.2 Hz), 7.95 (t, 1H, ^3J = 7.5 Hz), 8.21 (br.d, 1H, $^3J_{obs}$ = 7.2 Hz), 8.29 (br.d, 1H, $^3J_{obs}$ = 7.1 Hz), 8.36 (s, 1H), 9.21 (br.s, 1H), 9.75 (s, 2H); ^{13}C NMR ($CDCl_3$) δ 11.4 (2C), 12.1 (2C), 14.1 (2C), 15.9 (2C), 22.7 (2C), 26.2 (2C), 31.9 (1C), 32.3 (2C), 32.8 (2C), 96.2 (1C), 97.0 (2C), 109.6, 118.2, 119.8, 122.5, 122.9, 127.9, 129.5, 133.6, 134.4, 135.7, 136.6, 137.5, 138.3, 141.0, 144.6, 146.7, 146.8, 147.6, 153.6; aromatic carbon atoms were not integrated due to ambiguity in their assignment and eventual overlapping of some signals; MS (MALDI-TOF) calcd. for $C_{50}H_{54}N_6Zn$: 802.37, found 802.21 $[M]^+$.

Zn complex of 8-{3-[4,5,9,15,19,20-hexamethyl-10,14-dipentyl-21,22,23,24-tetraazapentacyclo[16.2.1.1^{3,6}.1^{8,11}.1^{13,16}]tetracosa-1,3,5,7,9,11(23),12,14,16,18(21),19-undecaen-2-yl]phenyl}-9-methyl-3,4,5,9-tetrahydro-1*H*-purine-2,6-dione (9) was obtained from porphyrin **2** (0.05 mmol, 38 mg), caffeine (1,3,7-trimethylpurine-2,6-dione) (0.1 mmol, 19 mg) in the presence of $Pd(OAc)_2$ (2.5 mg), $Cu(OAc)_2 \cdot 2H_2O$ (2 mg), PPh_3 (13 mg) and K_2CO_3 (14 mg) in 3 ml toluene. Eluent CH_2Cl_2/MeOH 100:1. Yield 41 mg (95%), dark-red crystalline powder. ^{1}H NMR ($CDCl_3$) δ 0.96 (t, 6H, ^3J = 7.3 Hz), 1.51 (sextet, 4H, ^3J = 7.3 Hz), 1.67 (quintet, 4H, ^3J = 7.1 Hz), 2.14 (br.s, 4H), 2.44 (s, 6H), 3.13 (br.s, 3H), 3.44 (s, 6H), 3.45 (s, 6H), 3.48 (br.s, 3H), 3.73 (br.s, 4H), 3.95 (br.s, 3H), 7.86 (br.t, 1H, $^3J_{obs}$ = 7.1 Hz), 8.07 (br.d, 1H, $^3J_{obs}$ = 7.0 Hz), 8.19 (br.d, 1H, $^3J_{obs}$ = 6.7 Hz), 8.32 (s, 1H), 9.49 (br.s, 1H), 9.86 (s, 1H), 9.87 (s, 1H); ^{13}C NMR ($CDCl_3$) δ 11.5 (2C), 12.1 (2C), 14.1 (2C), 15.9 (2C), 22.7 (2C), 26.3 (2C), 27.7 (1C), 29.7 (1C), 32.3 (2C), 32.9 (2C), 33.9 (1C), 96.5 (1C), 97.2 (2C), 108.5, 117.9, 127.7, 127.9, 128.9, 133.4, 134.8, 136.0, 137.3, 138.4, 141.3, 144.9, 146.5, 146.9, 147.9, 148.1, 148.2, 151.5, 151.9, 155.3; aromatic carbon atoms were not integrated due to ambiguity in their assignment and eventual overlapping of some signals; MS (MALDI-TOF) calcd. for $C_{48}H_{52}N_8O_2Zn$: 864.38, found 864.52. $[M]^+$.

Zn complex of 2,12-bis[4-(1,3-benzoxazol-2-yl)phenyl]-4,10,14,20-tetramethyl-5,9,15,19-tetrapropyl-21,22,23,24-tetraazapentacyclo[16.2.1.1^{3,6}.1^{8,11}.1^{13,16}]tetracosa-1(21),2,4,6,8(23),9,11,13,15,17,19-undecaene (13) was obtained from porphyrin **10** (0.05 mmol, 45 mg), benzoxazole (0.2 mmol, 24 mg) in the presence of $Pd(OAc)_2$ (2.5 mg), $Cu(OAc)_2 \cdot 2H_2O$ (2 mg), PPh_3 (13 mg) and K_2CO_3 (14 mg) in 3 ml toluene. Eluent CH_2Cl_2/MeOH 100:1. Yield 32 mg (65%), dark-red crystalline powder. ^{1}H NMR ($CDCl_3$) δ 1.18 (t, 12H, ^3J = 7.1 Hz), 2.13 (sextet, 8H, ^3J = 7.3 Hz), 2.42 (s, 12H), 3.86 (t, 8H, ^3J = 7.0 Hz), 7.37-7.42 (m, 4H), 7.64-7.68

(m, 2H), 7.78-7.82 (m, 2H), 8.21 (d, 4H, ^3J = 7.6 Hz), 8.55 (d, 4H, ^3J = 7.6 Hz), 10.06 (s, 2H); MS (MALDI-TOF) calcd. for $C_{62}H_{58}N_6O_2Zn$: 982.39, found 982.19. $[M]^+$.

Zn complex of 12-[4-(1,3-benzothiazol-2-yl)phenyl]-2-(4-bromophenyl)-4,10,14,20-tetramethyl-5,9,15,19-tetrapropyl-21,22,23,24-tetraazapentacyclo[16.2.1.1^{3,6}.1^{8,11}.1^{13,16}]tetracosa-1(21),2,4,6,8(23),9,11,13,15,17,19-undecaene (14) was obtained from porphyrin **10** (0.05 mmol, 45 mg), benzothiazole (0.2 mmol, 27 mg (29 μl)) in the presence of $Pd(OAc)_2$ (2.5 mg), $Cu(OAc)_2{\cdot}2H_2O$ (2 mg), PPh_3 (13 mg) and K_2CO_3 (14 mg) in 3 ml toluene. Eluent CH_2Cl_2/MeOH 100:1. Yield 31 mg (64%), dark-red crystalline powder. ^{1}H NMR ($CDCl_3$) δ 1.26 (br.s, 12H), 2.20 (br.s 8H), 2.47 (s, 6H), 2.51 (s, 6H), 3.91 (br.s, 8H), 7.45-7.60 (m, 3H), 7.90-7.99 (m, 3H), 8.13 (br.d, 2H, $^3J_{obs}$ = 7.3 Hz), 8.21 (br.d, 2H, $^3J_{obs}$ = 6.8 Hz), 8.49 (br.d, 2H, $^3J_{obs}$ = 6.1 Hz), 10.1 (s, 2H); MS (MALDI-TOF) calcd. for $C_{55}H_{54}BrN_5SZn$: 959.258, found 959.260. $[M]^+$.

Zn complex of 2-(4-bromophenyl)-4,10,14,20-tetramethyl-12-[4-(1-methyl-1*H*-benzimidazol-2-yl)phenyl]-5,9,15,19-tetrapropyl-21,22,23,24-tetraazapentacyclo[16.2.1.1^{3,6}.1^{8,11}.1^{13,16}]tetracosa-1(21),2,4,6,8(23),9,11,13,15,17,19-undecaene (15) was obtained from porphyrin **10** (0.05 mmol, 45 mg), 1-methylbenzimidazole (0.2 mmol, 26 mg) in the presence of $Pd(OAc)_2$ (2,5 mg), $Cu(OAc)_2{\cdot}2H_2O$ (2 mg), PPh_3 (13 mg) and K_2CO_3 (14 mg) in 3 ml toluene. Eluent CH_2Cl_2/MeOH 100:1. Yield 39 mg (83%), dark-red crystalline powder. ^{1}H NMR ($CDCl_3$) δ 1.26 (t, 12H, ^3J = 7.1 Hz), 2.21 (br.sextet, 8H, $^3J_{obs}$ = 6.2 Hz), 2.48 (s, 6H), 2.54 (s, 6H), 3.94 (br.s, 8H), 4.16 (s, 3H), 7.37-7.45 (m, 2H), 7.52-7.56 (m, 1H), 7.88 (br.d, 2H, $^3J_{obs}$ = 6.6 Hz), 7.91-7.99 (m, 3H), 8.17 (br.d, 2H, $^3J_{obs}$ = 6.3 Hz), 8.27 (br.d, 2H, $^3J_{obs}$ = 6.4 Hz), 10.17 (s, 2H); MS (MALDI-TOF) calcd. for $C_{56}H_{57}BrN_6Zn$: 956.31, found 956.00. $[M]^+$.

Zn complex of 2,12-bis[4-(1,3-benzoxazol-2-yl)phenyl]-4,10,14,20-tetramethyl-5,9,15,19-tetrapentyl-21,22,23,24-tetraazapentacyclo[16.2.1.1^{3,6}.1^{8,11}.1^{13,16}]tetracosa-1(21),2,4,6,8(23),9,11,13,15,17,19-undecaene (16) was obtained from porphyrin **11** (0.05 mmol, 51 mg), benzoxazole (0.125 mmol, 14 mg) in the presence of $Pd(dba)_2$ (6 mg), DavePhos (4 mg), and Cs_2CO_3 (33 mg) in 1.5 ml dioxane. Eluent CH_2Cl_2/MeOH 100:1. Yield 29 mg (52%), dark-red crystalline powder. ^{1}H NMR ($CDCl_3$) δ 0.95 (t, 12H, ^3J = 6.8 Hz), 1.53 (sextet, 8H, ^3J = 6.9 Hz), 1.71 (quintet, 8H, ^3J = 7.2 Hz), 2.16 (br.s, 8H), 2.49 (s, 12H), 3.91 (br.s, 8H), 7.45-7.50 (m, 4H), 7.70-7.75 (m, 2H), 7.89-7.93 (m, 2H), 8.26 (br.d, 4H, $^3J_{obs}$ = 7.2 Hz), 8.66 (br.d, 4H, $^3J_{obs}$ = 7.2 Hz), 10.14 (s, 2H); ^{13}C NMR ($CDCl_3$) δ 14.15 (4C), 15.6 (4C), 22.8 (4C), 26.7 (4C), 32.5

(4C), 33.0 (4C), 97.9 (2C), 110.7, 118.2, 120.2, 124.8, 125.3, 126.7, 127.0, 133.9, 137.7, 142.4, 143.9, 146.5, 147.3, 147.5, 151.0, 163.4; aromatic carbon atoms were not integrated due to ambiguity in their assignment and eventual overlapping of some signals; MS (MALDI-TOF) calcd. for $C_{70}H_{74}N_6O_2Zn$: 1094.52, found 1094.48. $[M]^+$.

Zn complex of 12-[3-(1,3-benzothiazol-2-yl)phenyl]-2-(4-bromophenyl)-4,10,14,20-tetramethyl-5,9,15,19-tetrapropyl-21,22,23,24-tetraazapentacyclo[16.2.1.13,6.18,11.113,16]tetracosa-1(21),2,4,6,8(23),9,11,13,15,17,19-undecaene (17) was obtained from porphyrin **12** (0.022 mmol, 20 mg), benzothiazole (0.088 mmol, 12 mg (13 μl)) in the presence of $Pd(OAc)_2$ (1 mg), $Cu(OAc)_2·2H_2O$ (1 mg), PPh_3 (6 mg) and K_2CO_3 (6 mg) in 3 ml toluene. Eluent CH_2Cl_2/MeOH 100:1. Yield 4 mg (19%), red crystalline powder. ^1H NMR ($CDCl_3$) δ 1.26 (t, 12H, ^3J = 7.7 Hz), 2.22 (sextet, 8H, ^3J = 7.6 Hz), 2.51 (s, 6H), 2.53 (s, 6H), 3.95 (t, 4H, ^3J = 7.3 Hz), 3.95 (t, 4H, ^3J = 7.6 Hz), 7.36-7.40 (m, 1H), 7.45-7.50 (m, 1H), 7.61 (t, 1H, ^3J = 7.7 Hz), 7.87-7.92 (m, 2H), 7.95 (d, 1H, ^3J = 8.4 Hz), 8.03-8.10 (m, 2H), 8.22 (d, 1H, ^3J = 7.7 Hz), 8.29 (s, 1H), 8.60 (d, 1H, ^3J = 7.7 Hz), 8.83 (s, 1H), 10.20 (s, 2H); MS (MALDI-TOF) calcd. for $C_{55}H_{54}BrN_5SZn$: 959.26, found 959.77. $[M]^+$.

Zn complex of 2,7,12,17-tetrakis[4-(1,3-benzoxazol-2-yl)phenyl]-21,22,23,24-tetraazapentacyclo[16.2.1.13,6.18,11.113,16]tetracosa-1(21),2,4,6,8(23),9,11,13,15,17,19-undecaene (19) was obtained from porphyrin **18** (0.05 mmol, 50 mg), benzoxazole (0.4 mmol, 48 mg) in the presence of $Pd(OAc)_2$ (5 mg), $Cu(OAc)_2·2H_2O$ (4 mg), PPh_3 (26 mg) and K_2CO_3 (35 mg) in 3 ml toluene. Eluent CH_2Cl_2/MeOH 100:1. Yield 8 mg (14%), dark-red crystalline powder. ^1H NMR ($CDCl_3$) δ 7.40-7.46 (m, 8H), 7.67-7.71 (m, 4H), 7.84-7.88 (m, 4H), 8.40 (d, 8H, ^3J = 7.7 Hz), 8.64 (d, 8H, ^3J = 7.7 Hz), 9.02 (s, 8H); MS (MALDI-TOF) calcd. for $C_{72}H_{40}N_8O_4Zn$: 1144.25, found 1144.22. $[M]^+$.

Zn complex of 2,12,17-tris[4-(1,3-benzoxazol-2-yl)phenyl]-7-bromo-21,22,23,24-tetraazapentacyclo[16.2.1.1^{3,6}.1^{8,11}.1^{13,16}]tetracosa-1(21),2,4,6,8(23),9,11,13,15,17,19-undecaene (20) was obtained from porphyrin **18** (0.05 mmol, 50 mg), benzoxazole (0.4 mmol, 48 mg) in the presence of $Pd(OAc)_2$ (5 mg), $Cu(OAc)_2·2H_2O$ (4 mg), PPh_3 (26 mg) and K_2CO_3 (35 mg) in 3 ml toluene. Eluent CH_2Cl_2/MeOH 50:1. Yield 44 mg (80%), dark-red crystalline powder. ^1H NMR ($CDCl_3$) δ 7.36-7.42 (m, 6H), 7.65 (br.s, 3H), 7.72 (br.s, 3H), 7.88 (d, 2H, ^3J = 8.2 Hz), 8.06 (d, 2H, ^3J = 8.2 Hz), 8.36 (br.d, 6H, $^3J_{obs}$ = 6.5 Hz), 8.54 (br.d, 6H, $^3J_{obs}$ = 6.5 Hz), 8.93-9.01 (m, 8H); MS (MALDI-TOF) calcd. for $C_{65}H_{36}BrN_7O_3Zn$: 1105.14, found 1105.05 $[M]^+$.

References

1. Ukai, T.; Kawazura, H.; Ishii, Y.; Bonnet, J. J.; Ibers, J. A. *J. Organomet. Chem.*, **1974**, *65*, 253-266.

2. Adler, A. D.; Longo, F. R.; Finarelli, J. D. *J. Org. Chem*., **1967**, *32,* 476.

3. Mikhalitsyna, E. A.; Tyurin, V. S.; Nefedov, S. E.; Syrbu, S. A.; Semeikin, A. S.; Koifman, O. I.; Beletskaya, I. P. *Eur. J. Inorg. Chem.*, **2012**, 5979-5990.

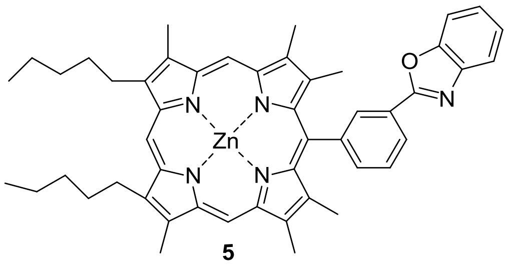

5

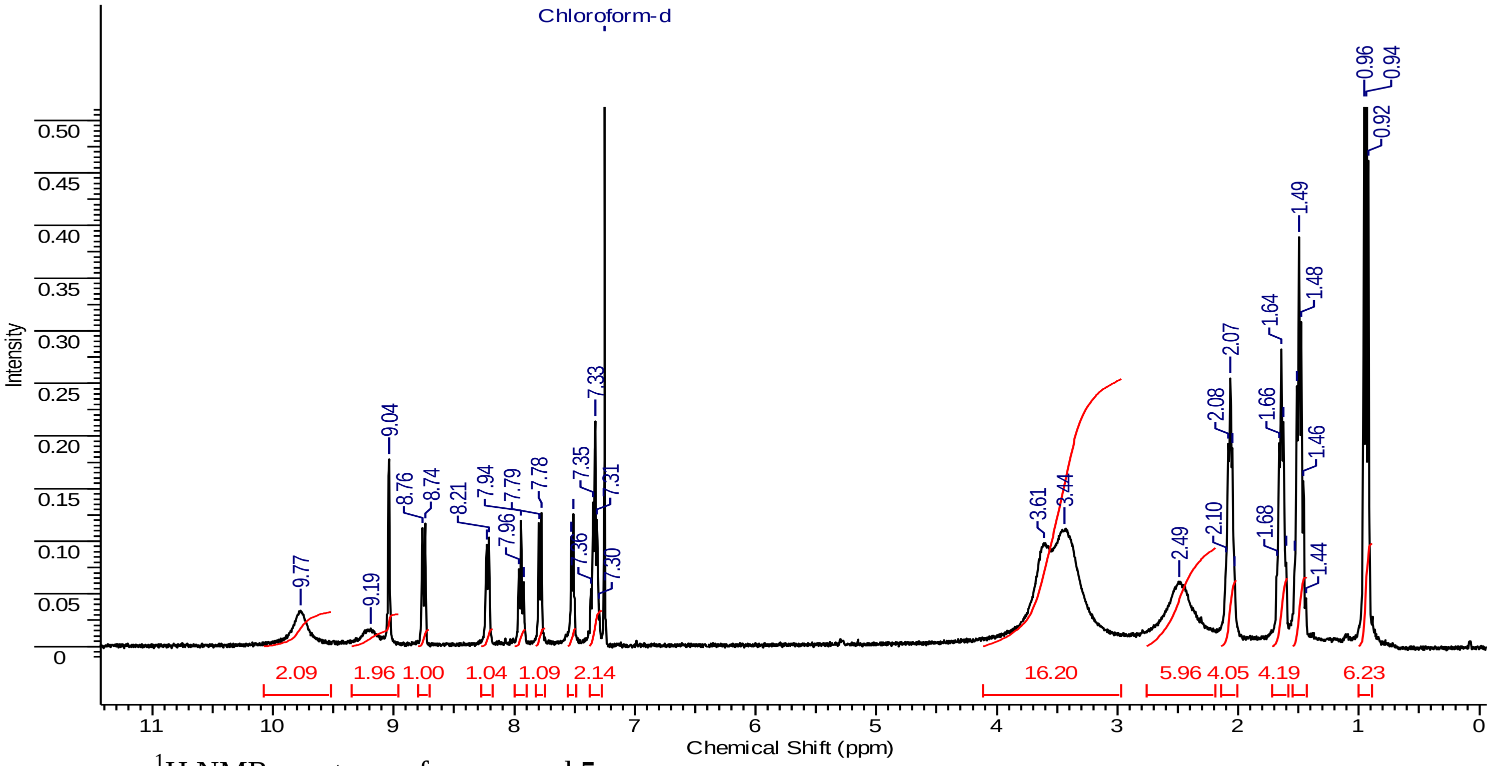

^{1}H NMR spectrum of compound **5**

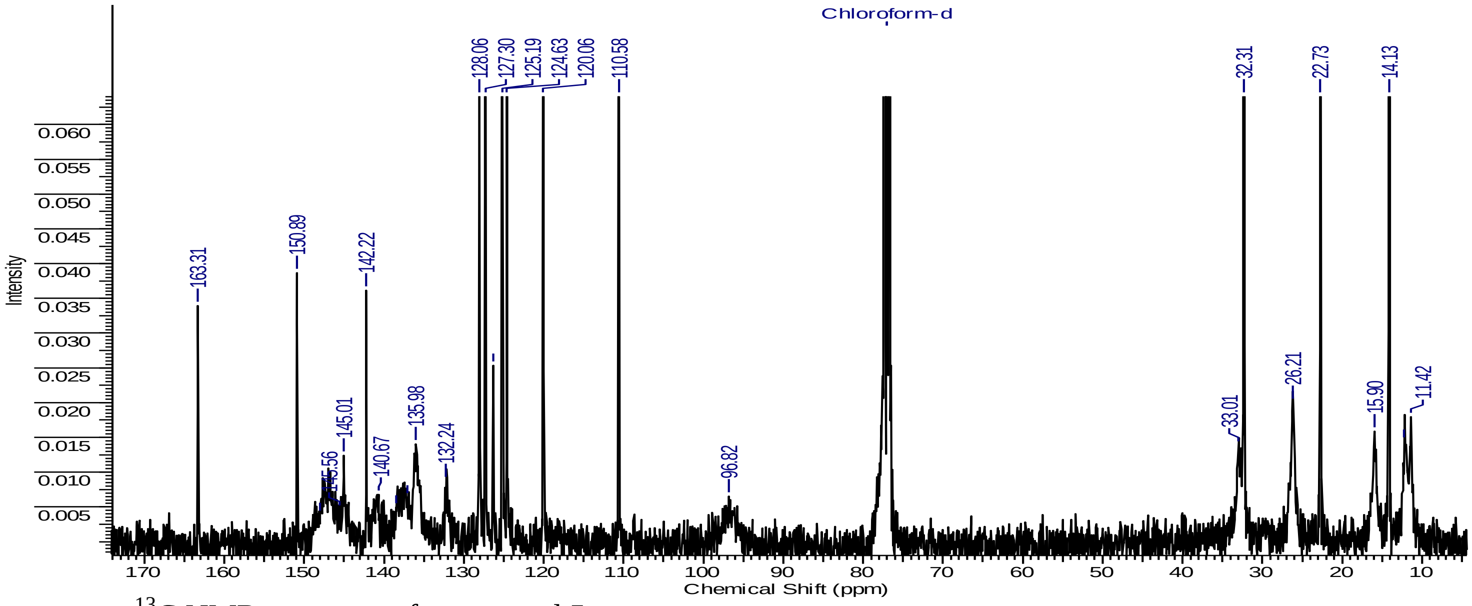

^{13}C NMR spectrum of compound **5**

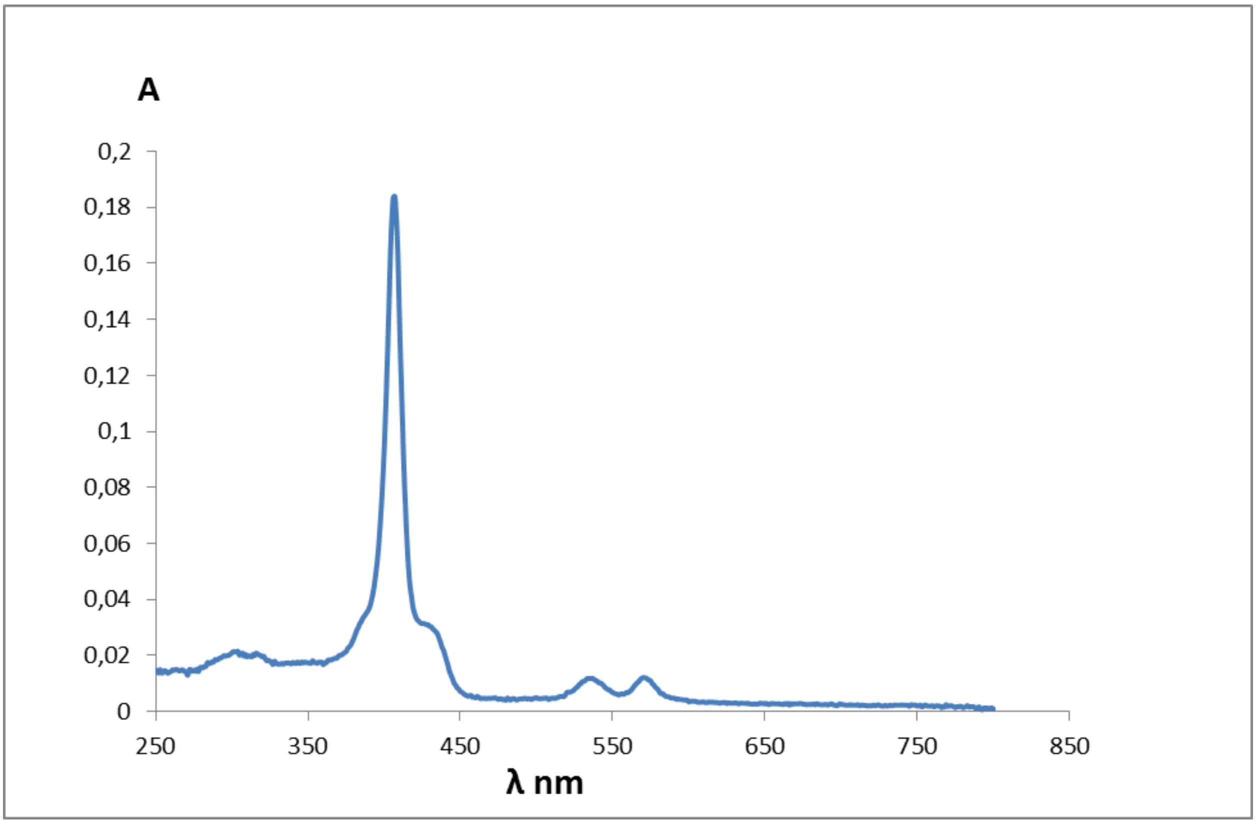

Absorbance spectrum of compound **5**

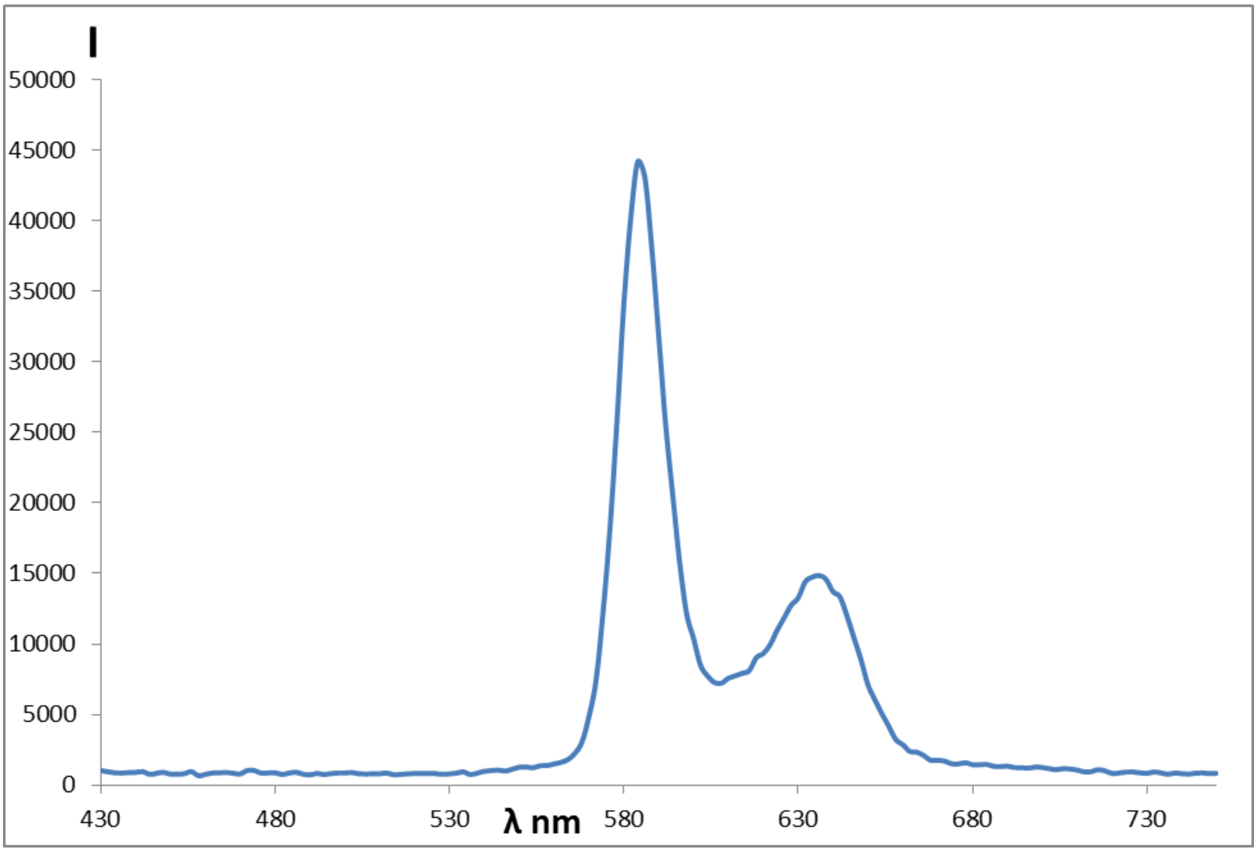

Fluorescence spectrum of compound **5**

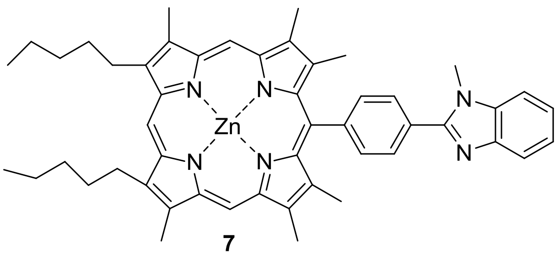

7

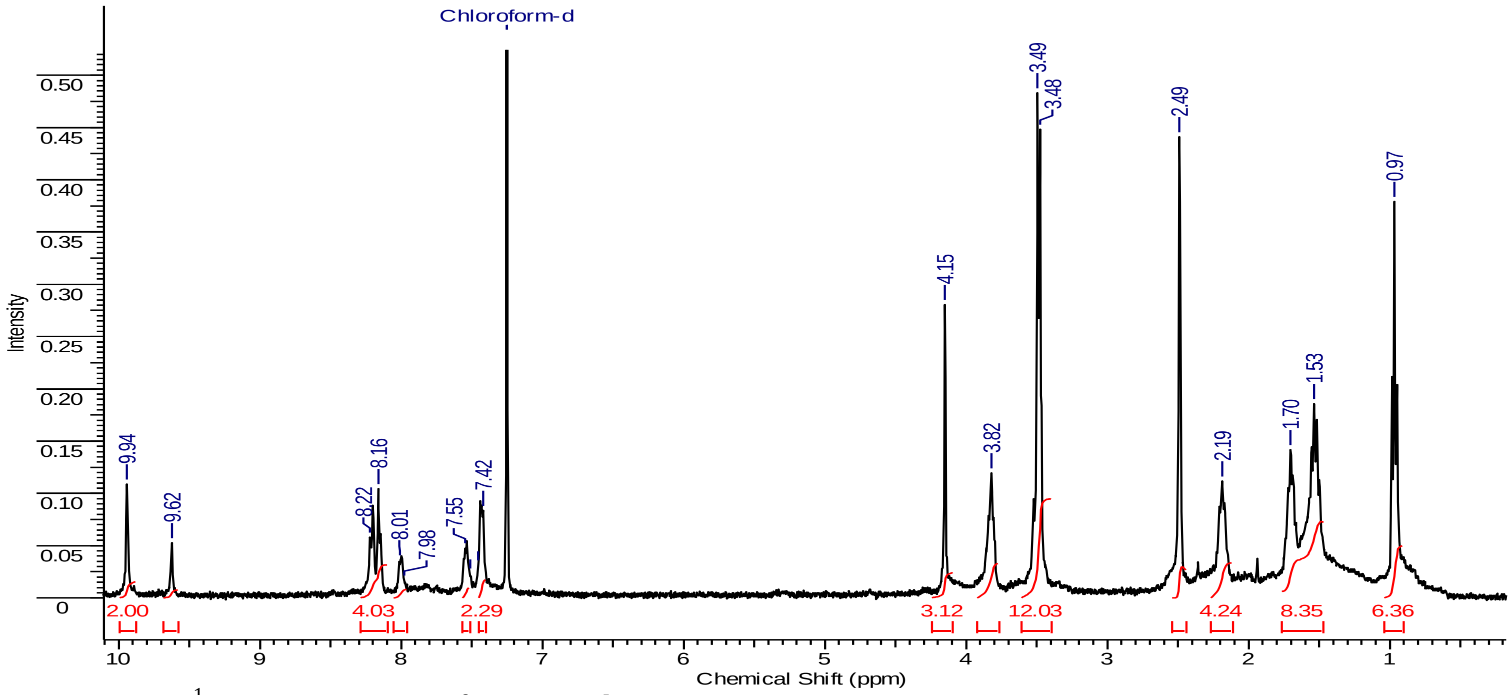

^{1}H NMR spectrum of compound **7**

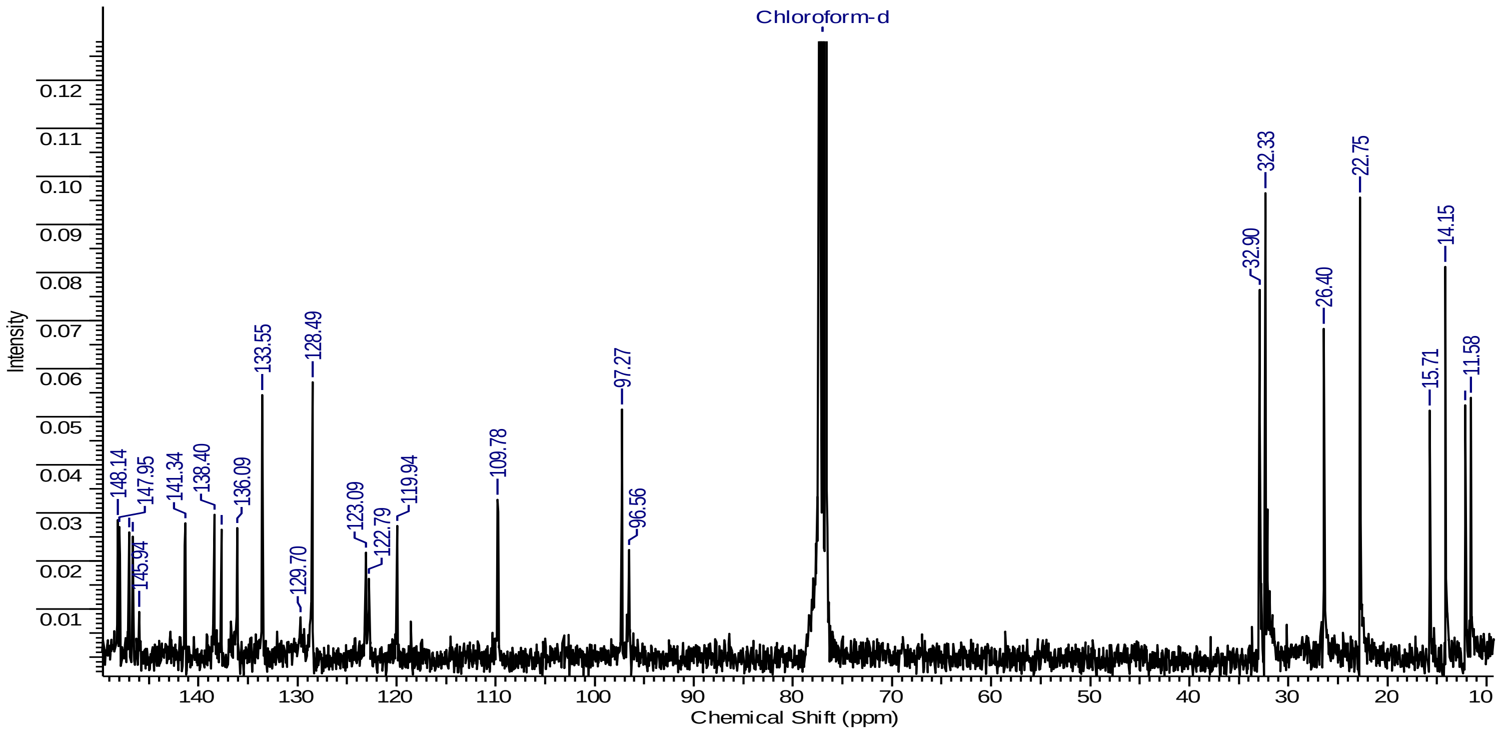

^{13}C NMR spectrum of compound **7**

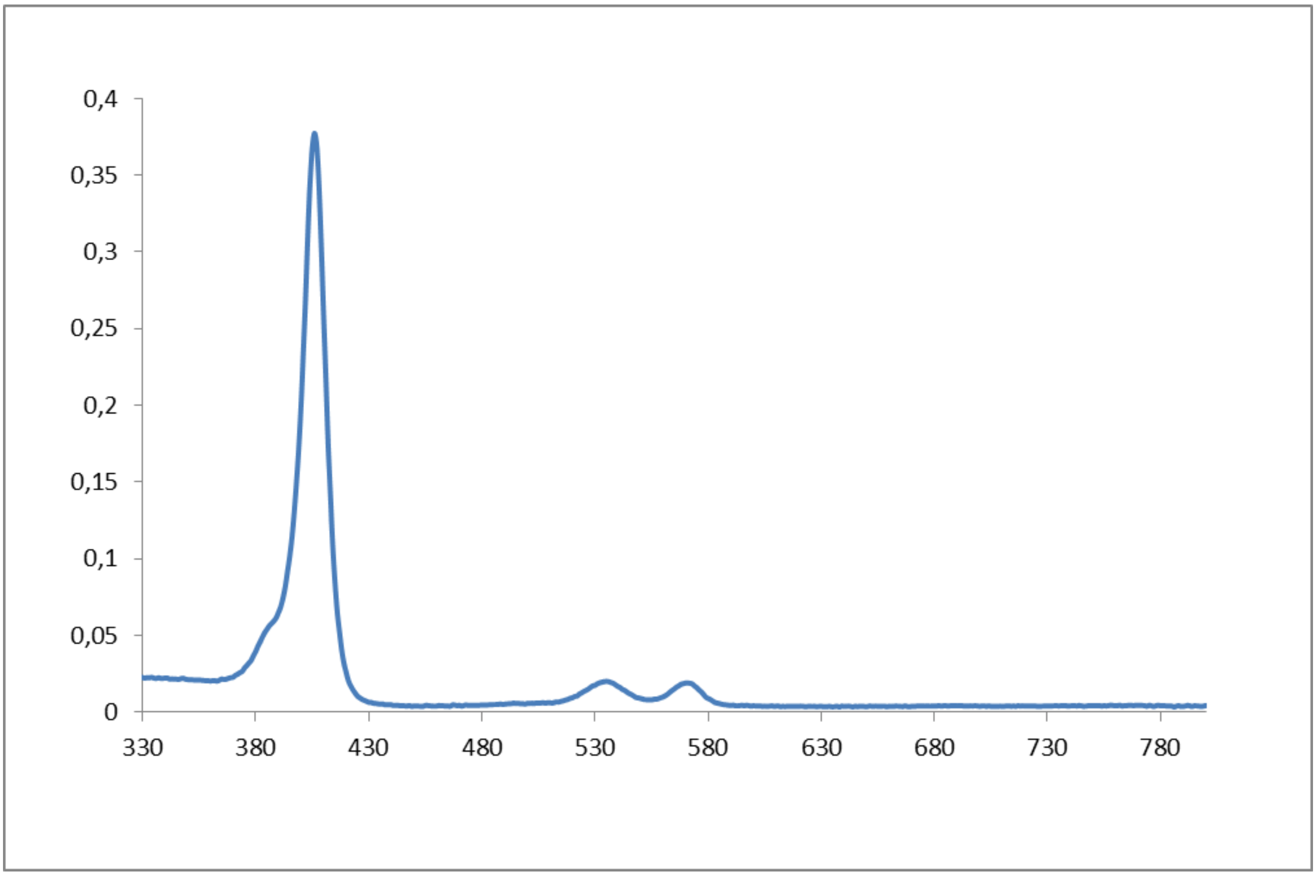

Absorbance spectrum of compound **7**

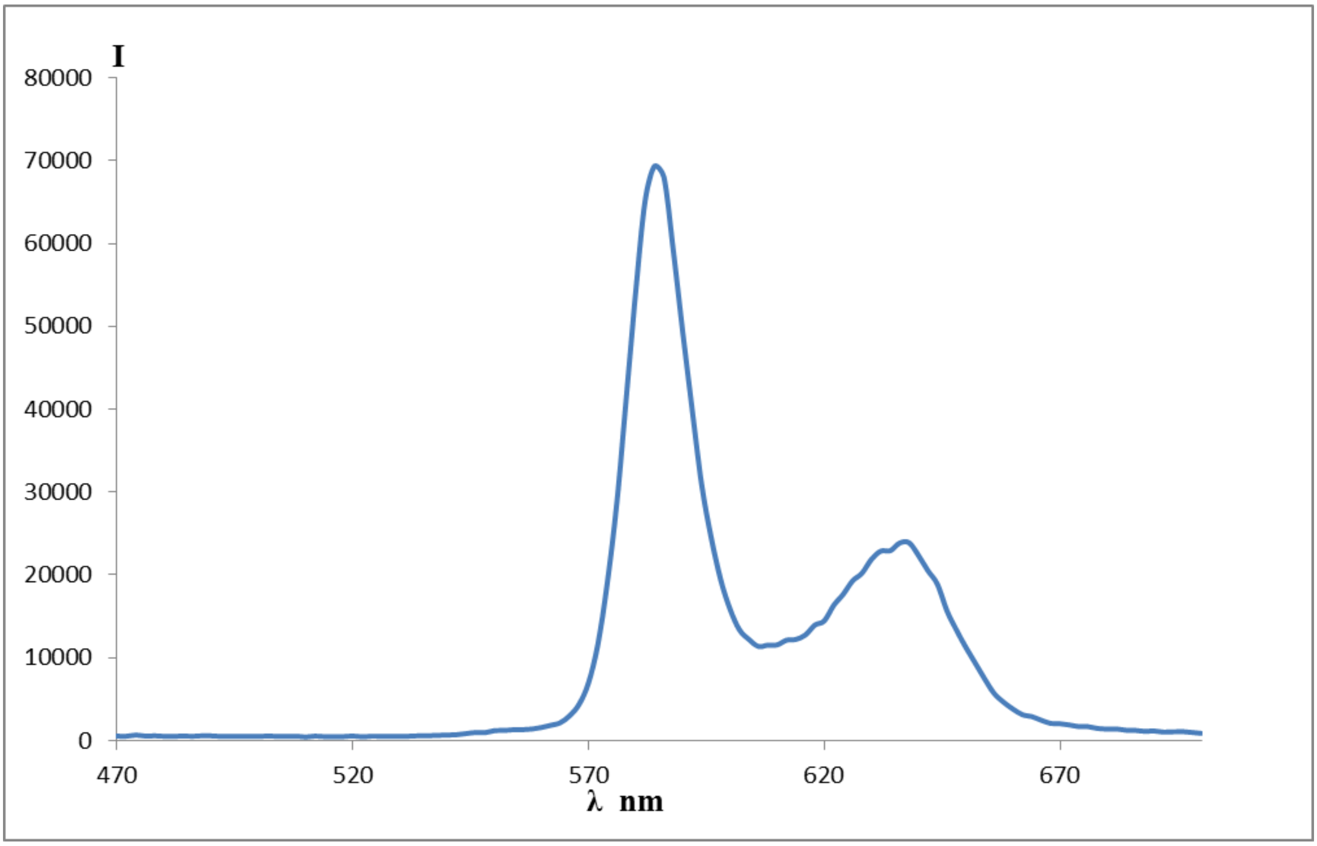

Fluorescence spectrum of compound **7**

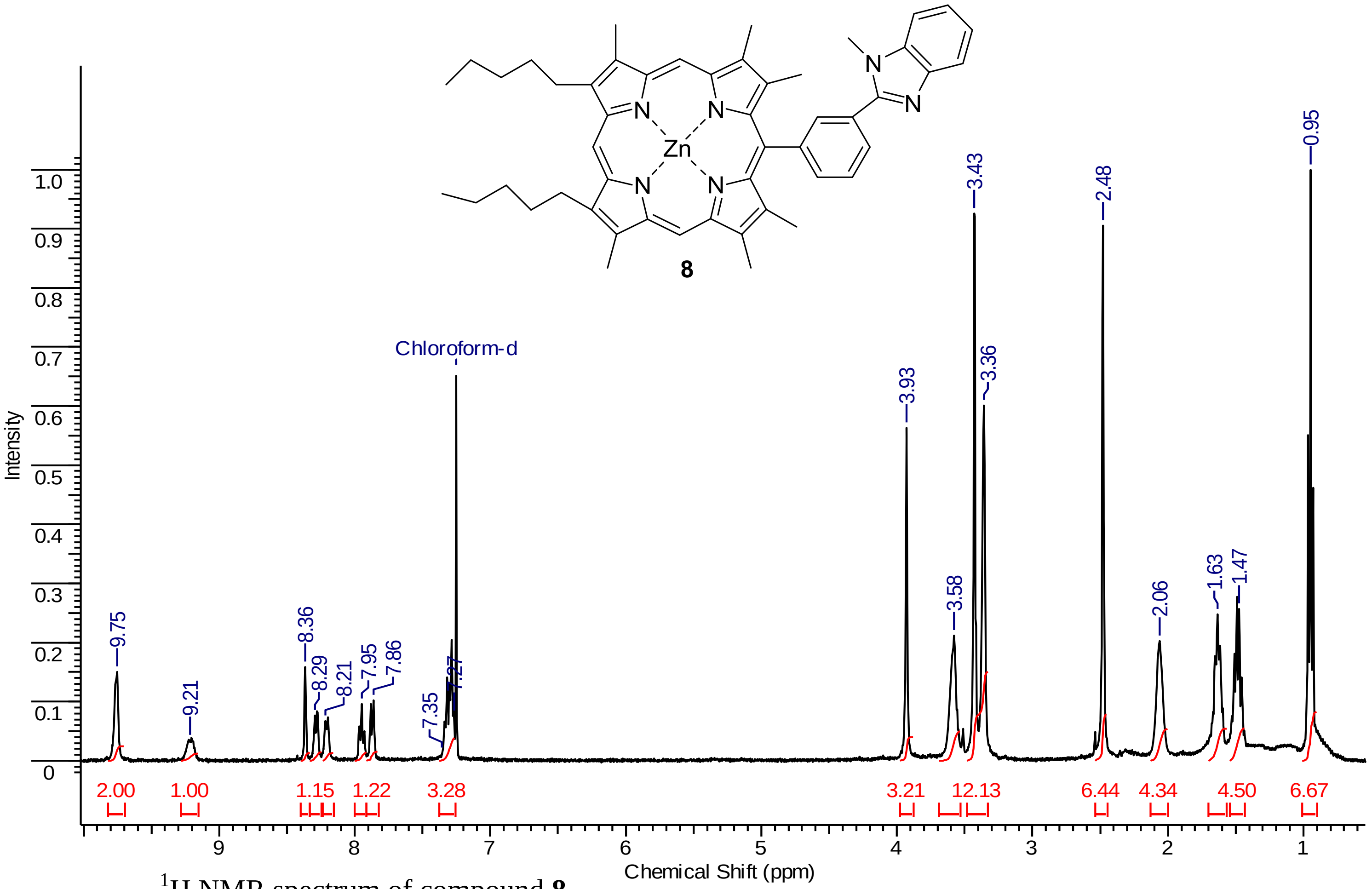

^{1}H NMR spectrum of compound **8**

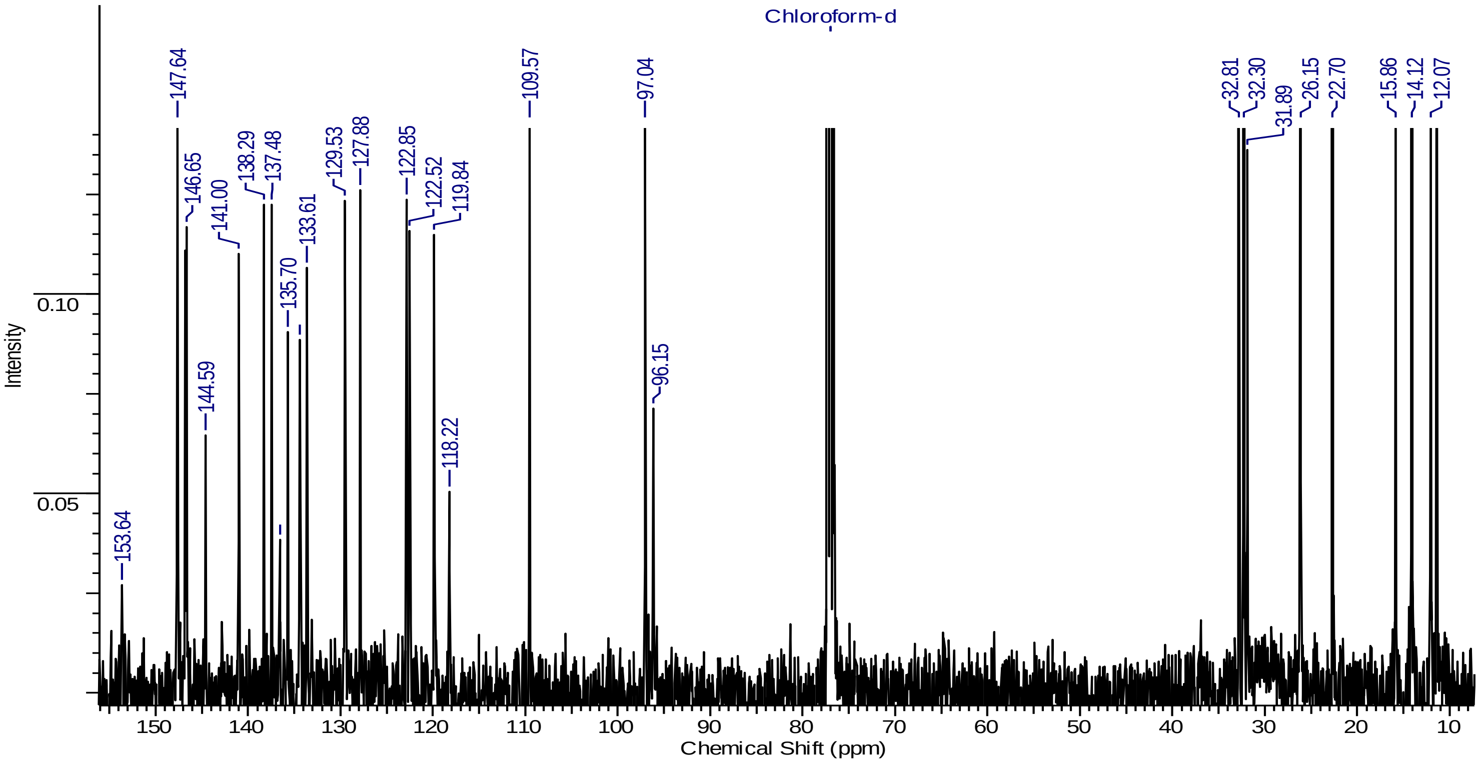

^{13}C NMR spectrum of compound **8**

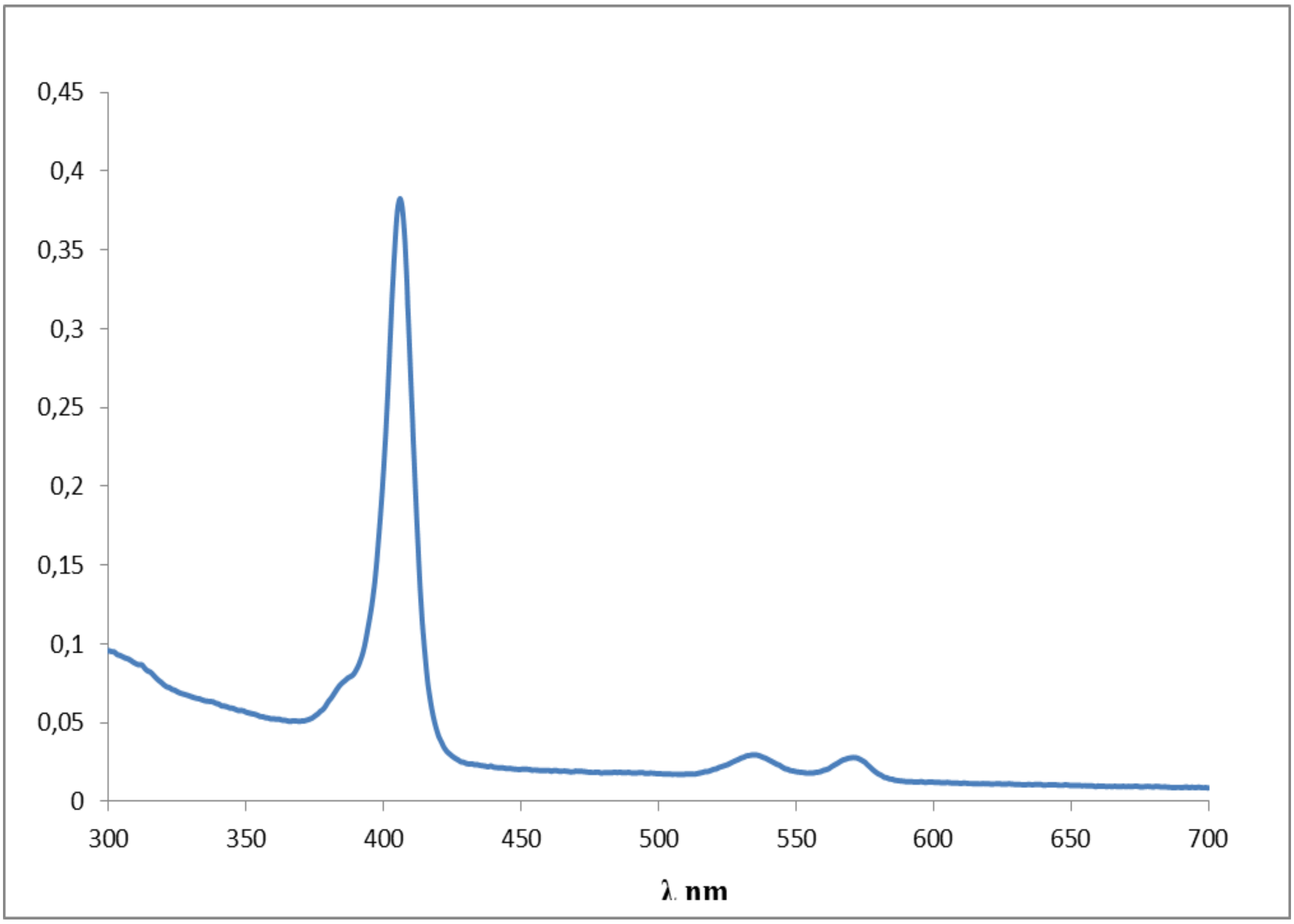

Absorbance spectrum of compound **8**

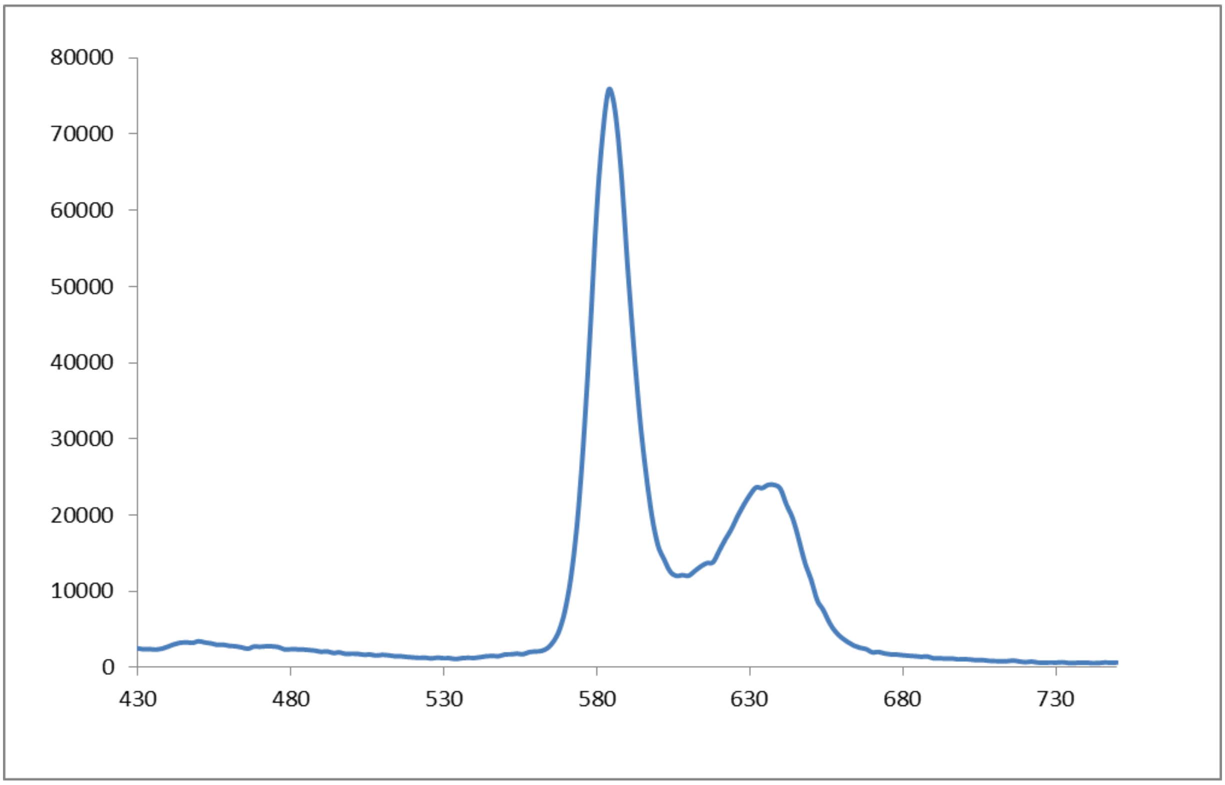

Fluorescence spectrum of compound **8**

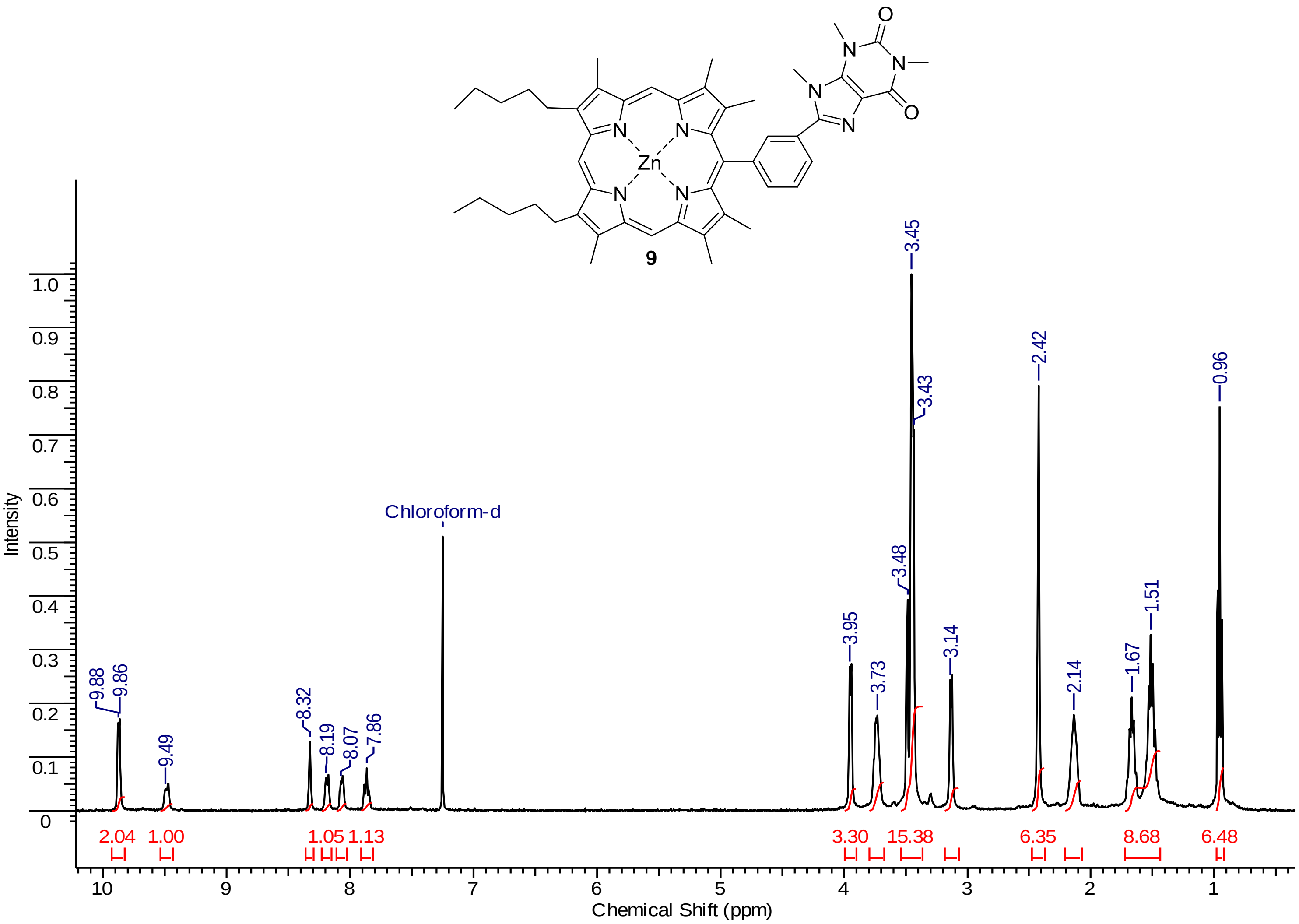

^{1}H NMR spectrum of compound **9**

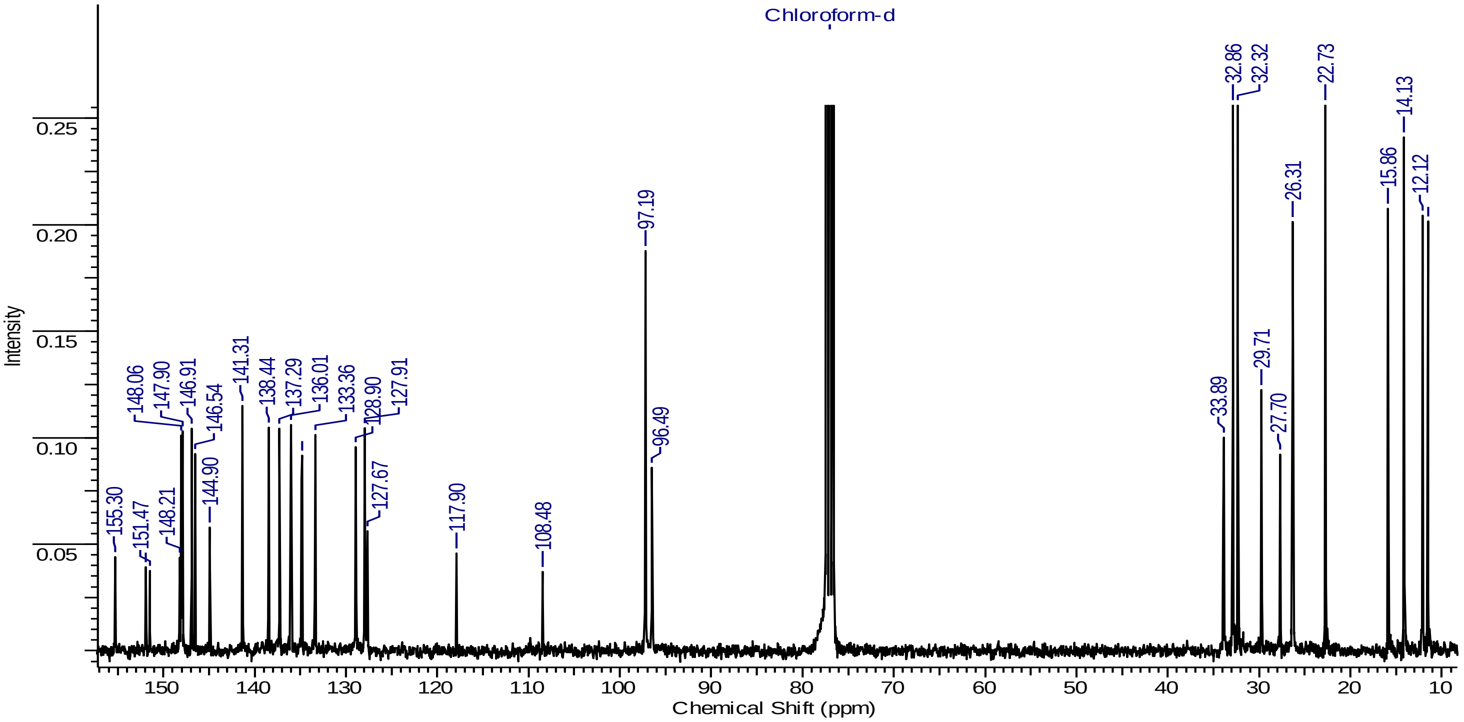

^{13}C NMR spectrum of compound **9**

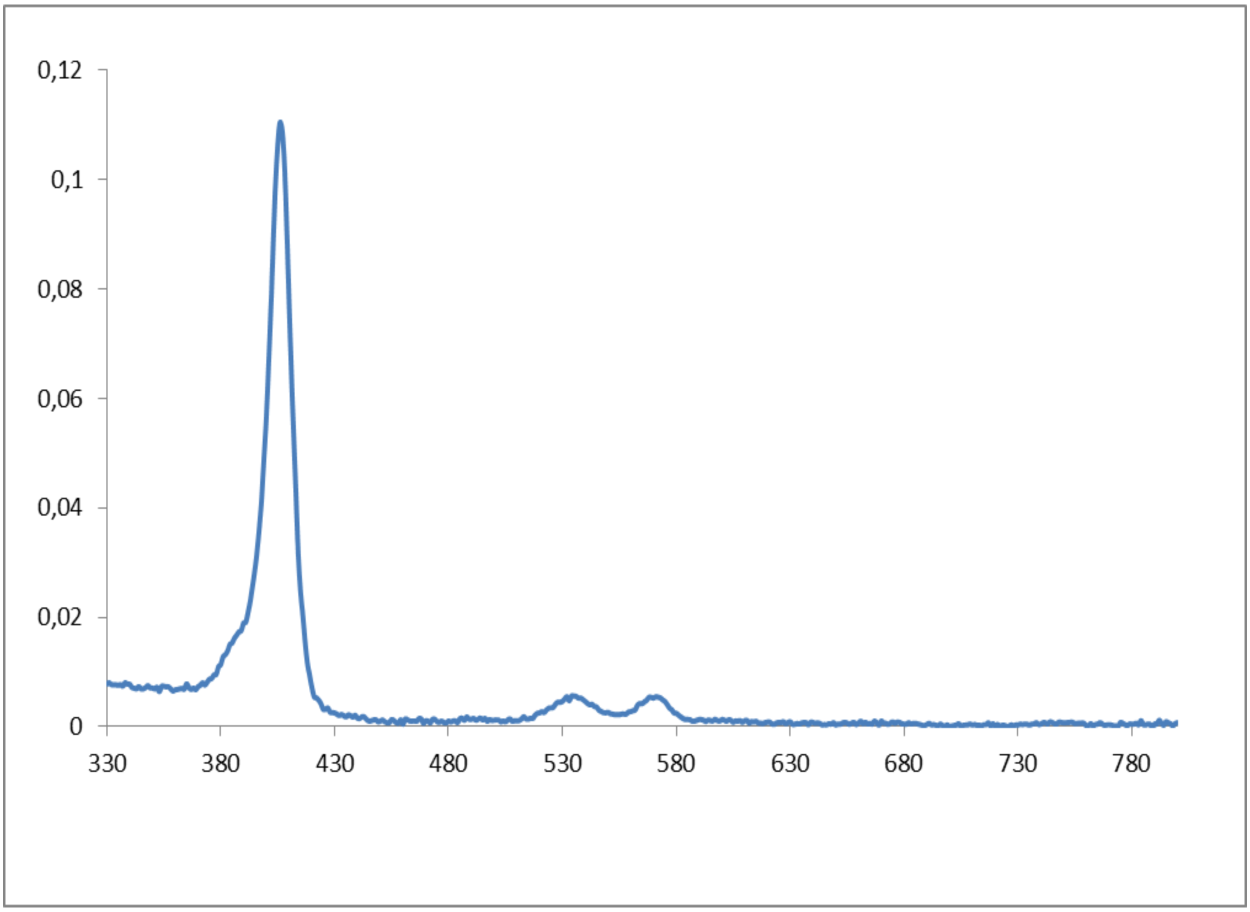

Absorbance spectrum of compound **9**

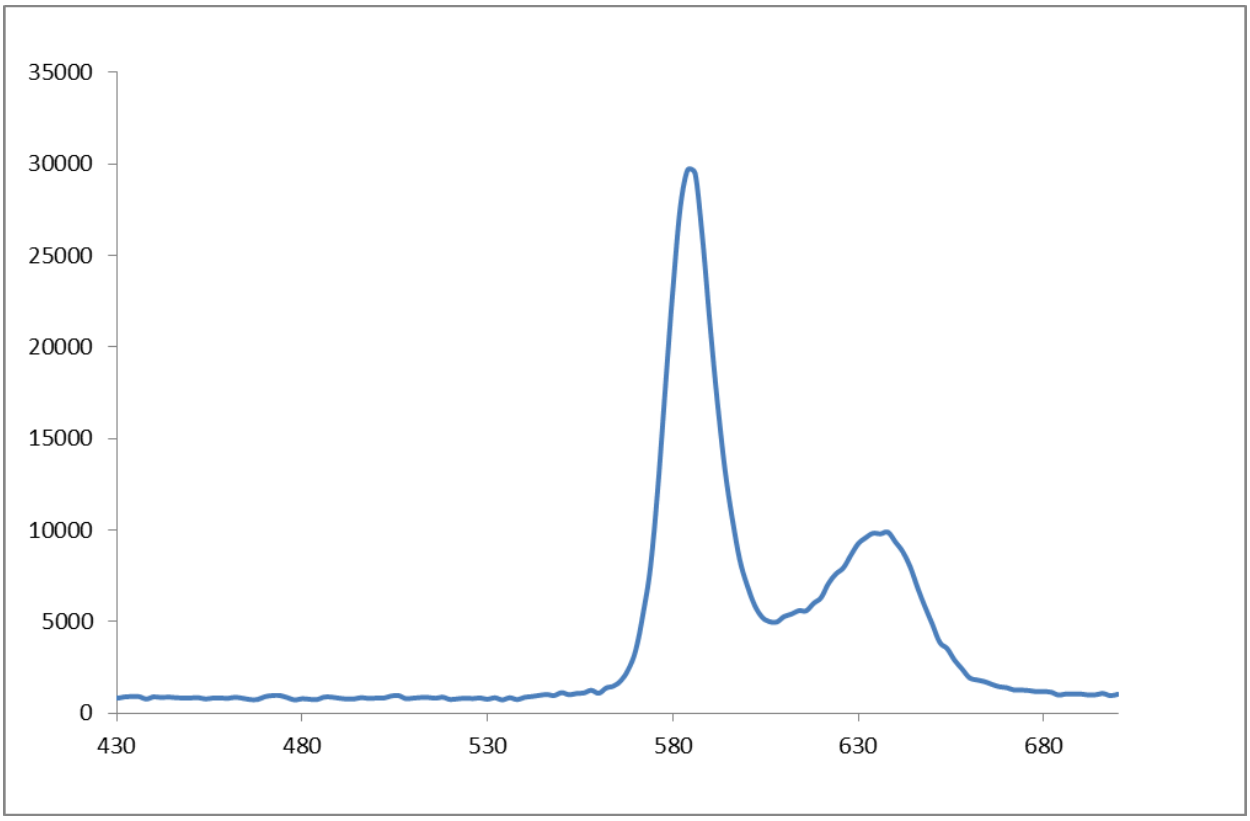

Fluorescence spectrum of compound **9**

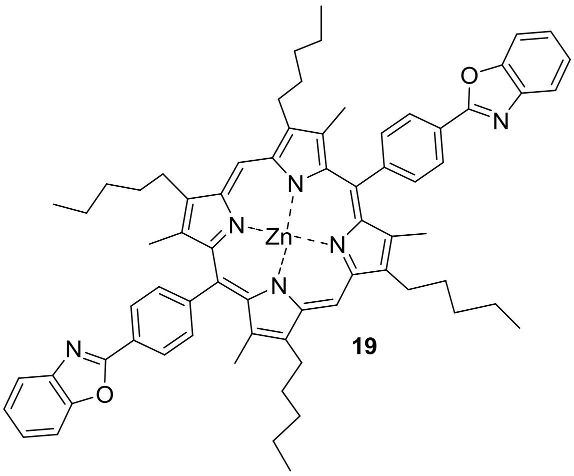

19

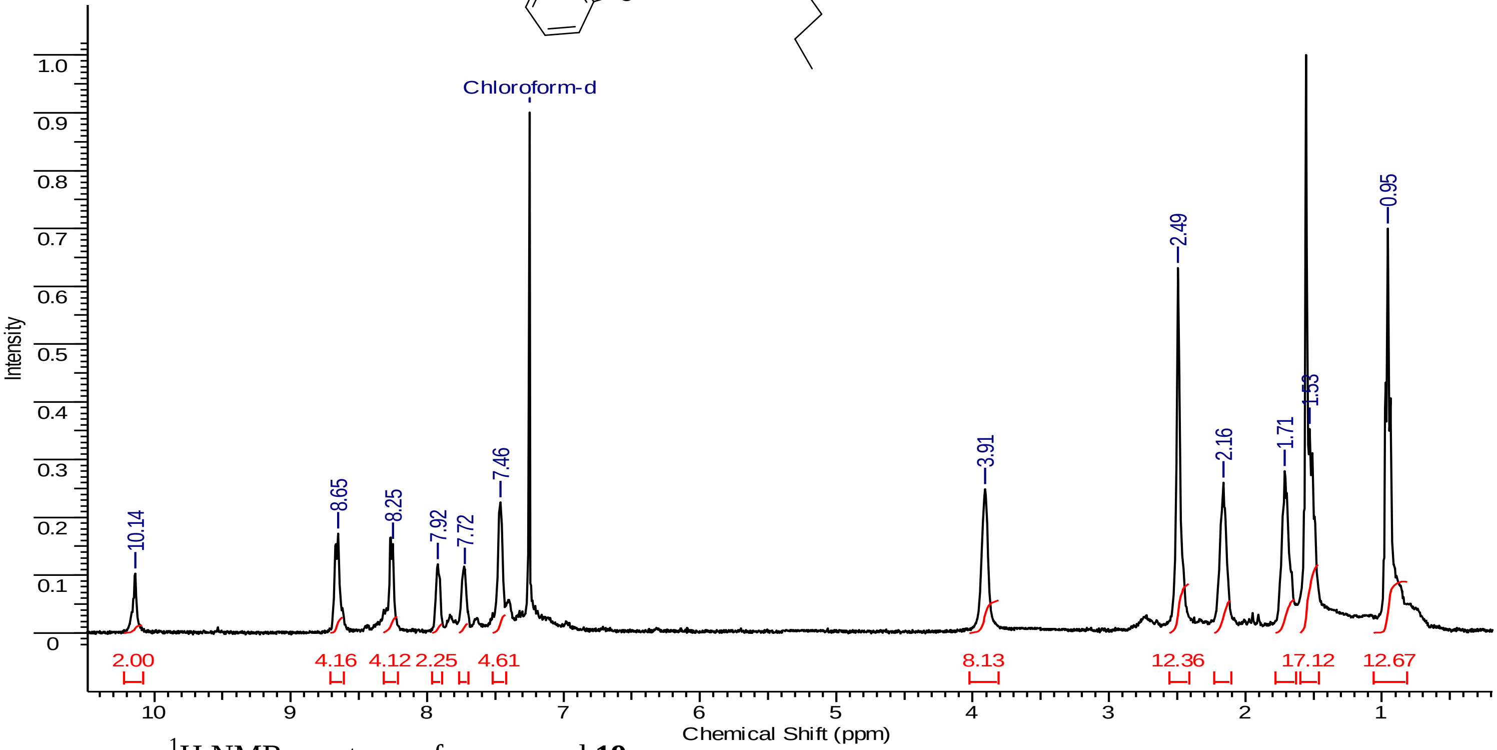

^{1}H NMR spectrum of compound **19**

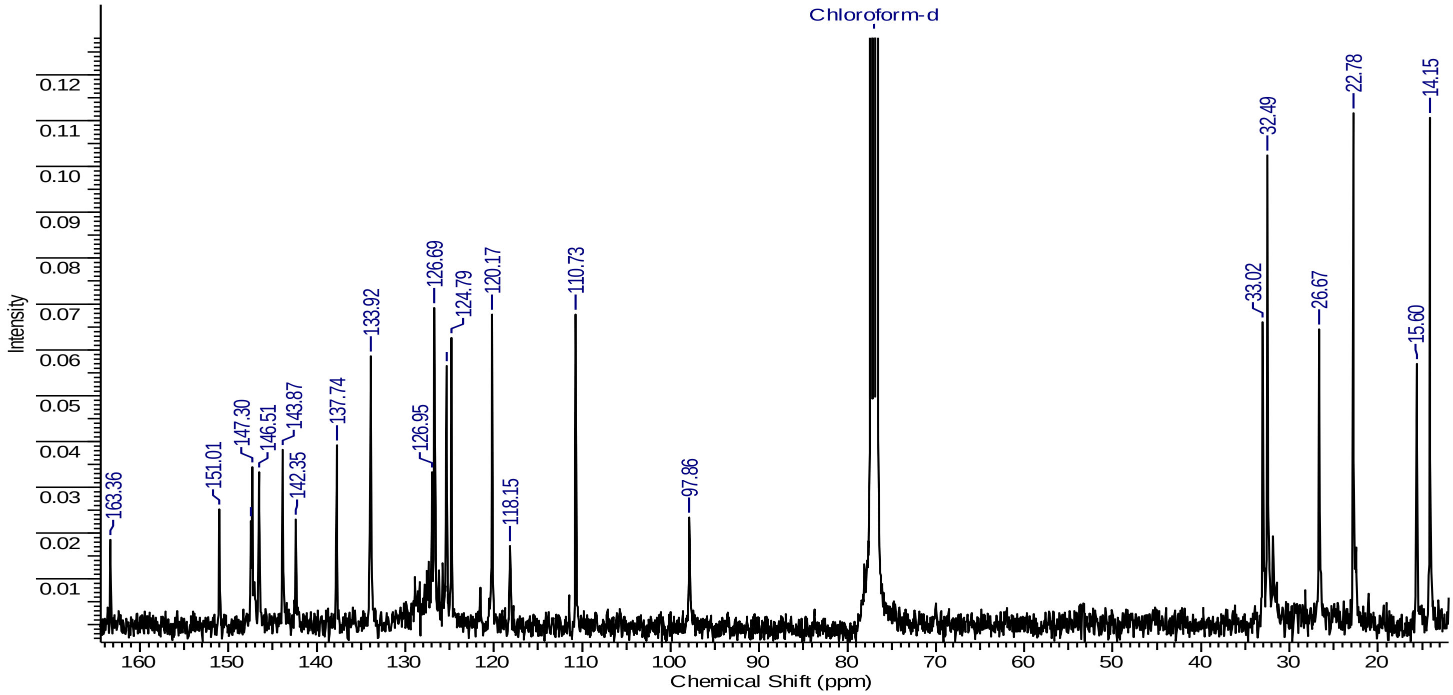

^{13}C NMR spectrum of compound **19**

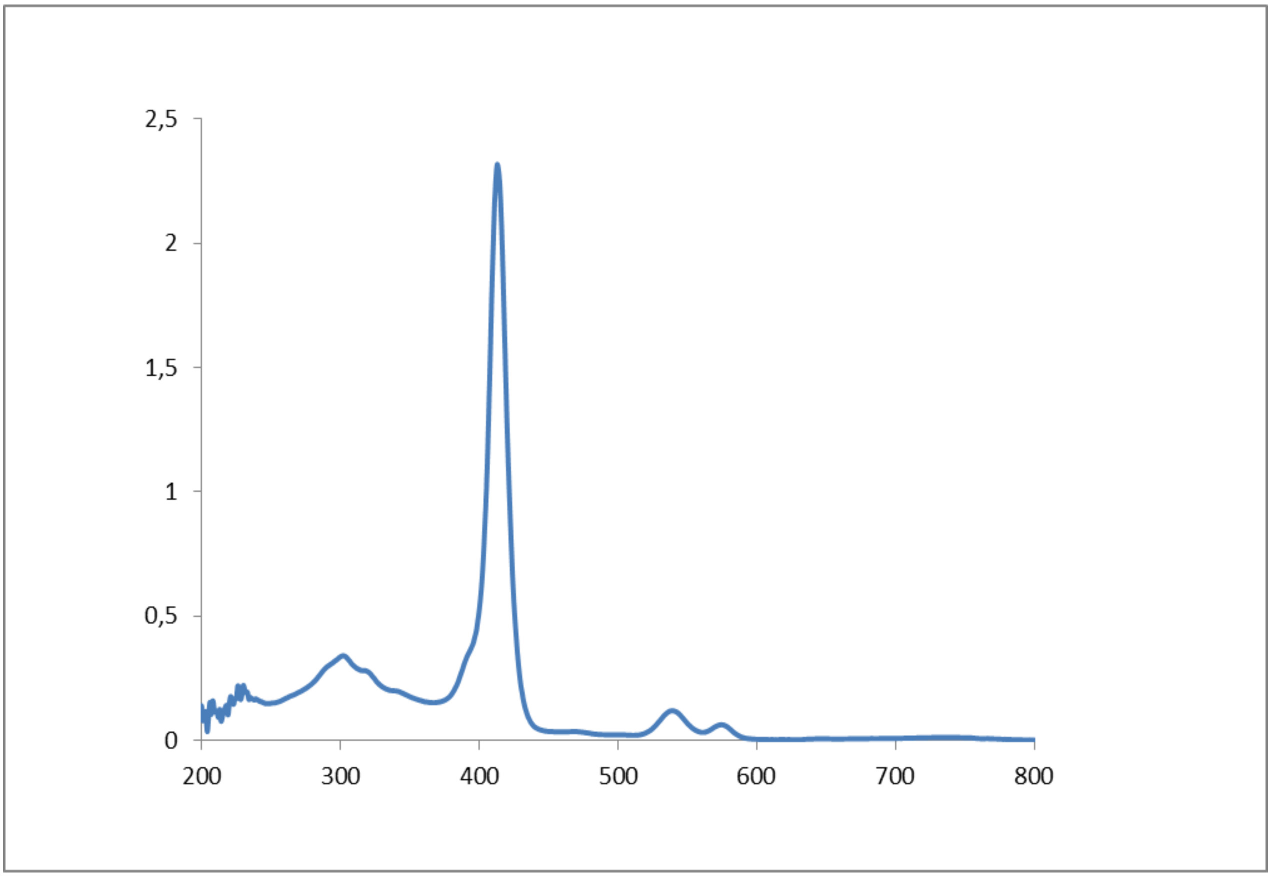

Absorbance spectrum of compound **19**

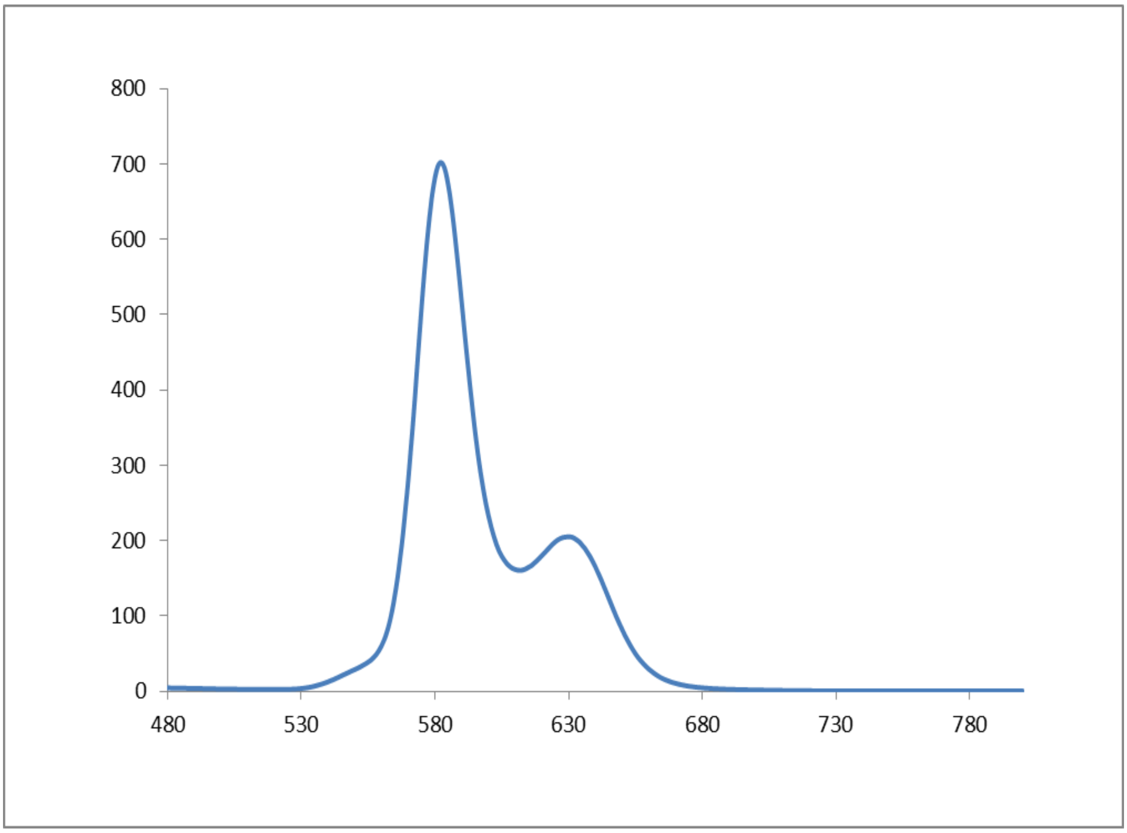

Fluorescence spectrum of compound **19**

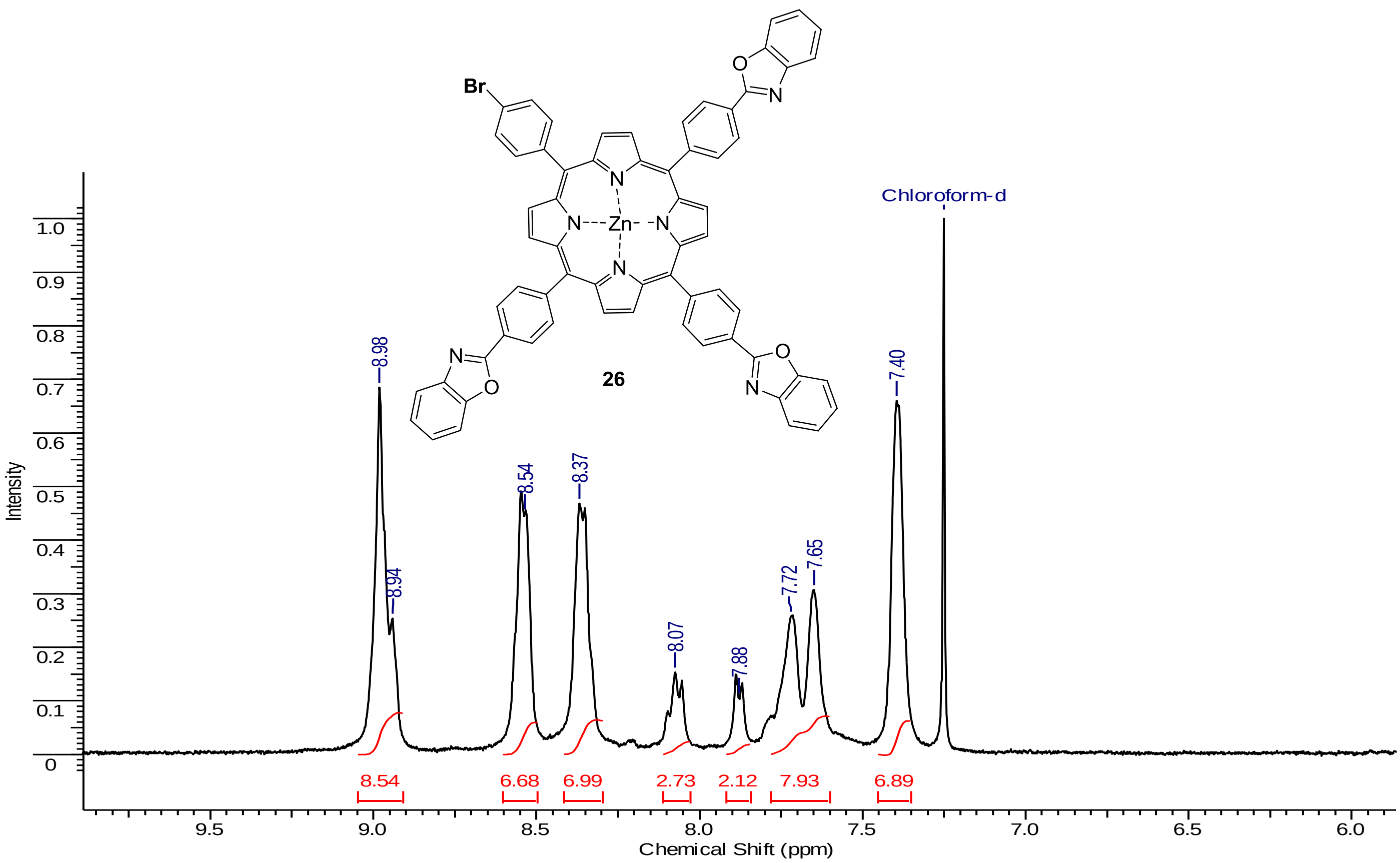

^{1}H NMR spectrum of compound **26**

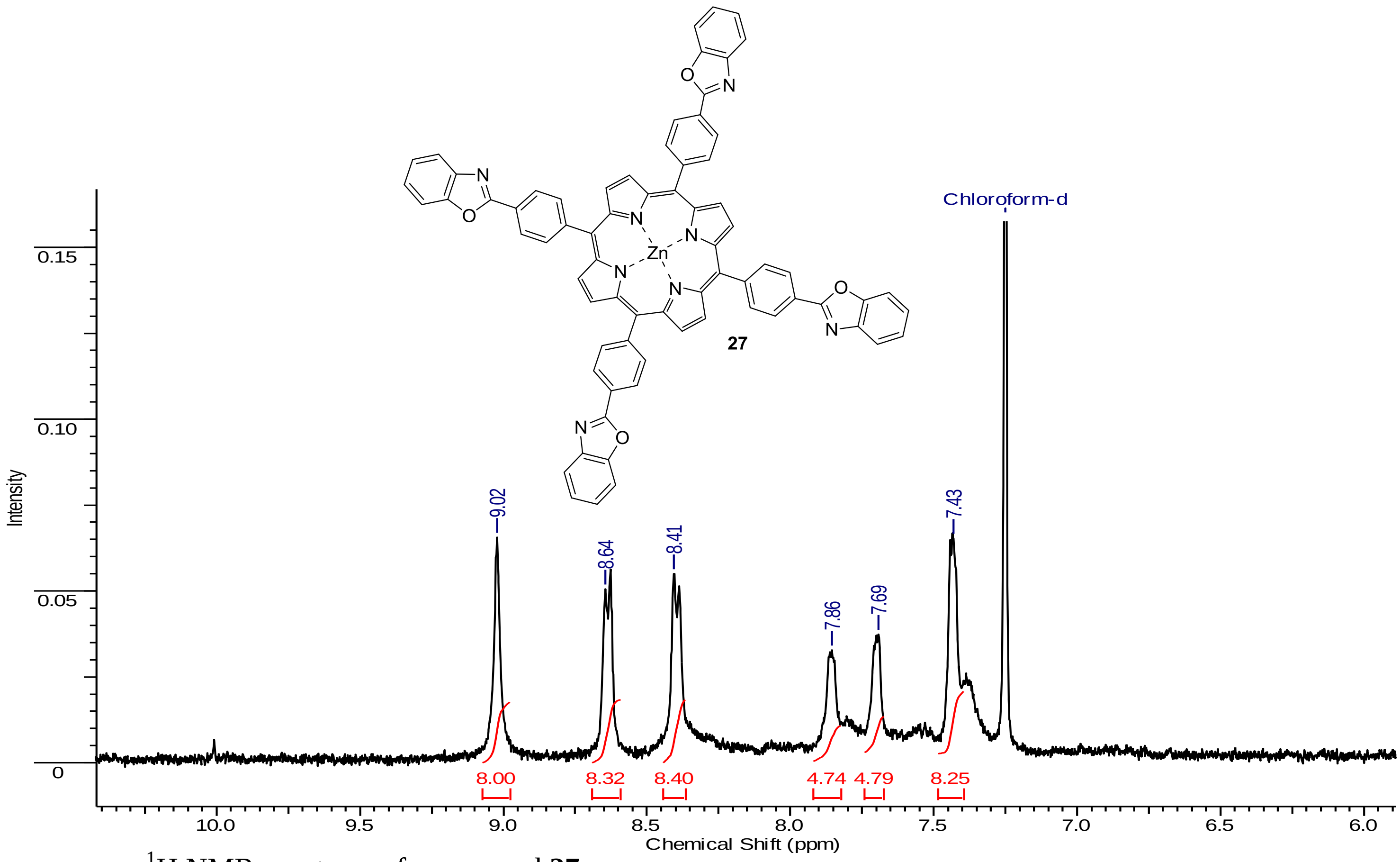

^{1}H NMR spectrum of compound **27**
